# Supplementary material for: MIND Pattern Nutritional Intervention Modulates Mediterranean Diet Adherence and Gut Microbiota in Alzheimer’s Disease: An Observational Case–Control Study
Source: Nutrients. 2026 Jan 7;18(2):193. doi: 10.3390/nu18020193 (PMC12844742; doi:10.3390/nu18020193)
Supplement: Supplementary file 1 [file nutrients-18-00193-s001.zip › nutrients-4031261-supplementary.pdf]

Supplementary Materials

Table S1 - Distribution of gut microbiota species and their relative abundances

Species	ALZ-T0	ALZ-T1	CTRL-T0	CTRL-T1
(Actinomycetota) *Actinomyces graevenitzii*	0,0003 ± 0,0018	0,0046 ± 0,0255	0,0013 ± 0,005	0,0003 ± 0,0018
(Actinomycetota) *Actinomyces israelii*	0,0016 ± 0,0091	0,0026 ± 0,0104	0 ± 0	0 ± 0
(Actinomycetota) *Actinomyces johnsonii*	0 ± 0	0 ± 0	0 ± 0	0 ± 0
(Actinomycetota) *Actinomyces massiliensis*	0 ± 0	0 ± 0	0 ± 0	0,0006 ± 0,0036
(Actinomycetota) *Actinomyces naeslundii*	0,0043 ± 0,0169	0,0026 ± 0,0104	0 ± 0	0,0006 ± 0,0036
(Actinomycetota) *Schaalia odontolytica*	0,0193 ± 0,046	0 ± 0	0 ± 0	0 ± 0
(Actinomycetota) *Actinomyces oris*	0,0103 ± 0,0418	0,0053 ± 0,0201	0,0013 ± 0,0073	0,009 ± 0,0492
(Actinomycetota) *Actinomyces radicidentis*	0,002 ± 0,0109	0 ± 0	0 ± 0	0 ± 0
(Actinomycetota) *Actinomyces turicensis*	0 ± 0	0 ± 0	0 ± 0	0 ± 0
(Actinomycetota) *Actinomyces viscosus*	0,002 ± 0,0109	0,0133 ± 0,0675	0 ± 0	0,002 ± 0,0109
(Actinomycetota) *Actinotignum schaalii*	0 ± 0	0 ± 0	0 ± 0	0 ± 0
(Actinomycetota) *Adlercreutzia equolifaciens*	0,0028 ± 0,0121	0,0783 ± 0,1275	0,0081 ± 0,0254	0,0206 ± 0,0459
(Actinomycetota) *Adlercreutzia muris*	0,0043 ± 0,0165	0,0586 ± 0,1003	0,0176 ± 0,0503	0,0154 ± 0,0521
(Actinomycetota) *Alloscardovia omnicolens*	0 ± 0	0,002 ± 0,0076	0 ± 0	0 ± 0
(Actinomycetota) *Aquihabitans daechungensis*	0 ± 0	0 ± 0	0 ± 0	0 ± 0
(Actinomycetota) *Arcanobacterium canis*	0 ± 0	0 ± 0	0 ± 0	0 ± 0
(Actinomycetota) *Arthrobacter agilis*	0 ± 0	0 ± 0	0 ± 0	0 ± 0
(Actinomycetota) *Arthrobacter bussei*	0 ± 0	0 ± 0	0 ± 0	0 ± 0
(Actinomycetota) *Arthrobacter citreus*	0,0434 ± 0,1258	0,3906 ± 0,419	0,2211 ± 0,3176	0,2968 ± 0,7054
(Actinomycetota) *Arthrobacter cryoconiti*	0 ± 0	0 ± 0	0 ± 0	0 ± 0
(Actinomycetota) *Arthrobacter luteolus*	0 ± 0	0,0036 ± 0,0099	0 ± 0	0 ± 0
(Actinomycetota) *Asaccharobacter celatus*	0,0503 ± 0,0955	0 ± 0	0,033 ± 0,0934	0,0656 ± 0,2513
(Actinomycetota) *Atopobium deltae*	0 ± 0	0 ± 0	0 ± 0	0 ± 0
(Actinomycetota) Atopobium minutum	0 ± 0	0 ± 0	0 ± 0	0 ± 0
(Actinomycetota) *Atopobium vaginae*	0 ± 0	0 ± 0	0 ± 0	0 ± 0
(Actinomycetota) *Bifidobacterium adolescentis*	4,9306 ± 10,6079	1,5323 ± 3,8186	1,2325 ± 1,6988	1,6801 ± 3,1056
(Actinomycetota) *Bifidobacterium Angulatum*	0,135 ± 0,5248	0 ± 0	0,0003 ± 0,0018	0 ± 0
(Actinomycetota*) Bifidobacterium animalis*	0,0026 ± 0,0146	0,0273 ± 0,0943	0,1929 ± 0,6768	0,1486 ± 0,5657
(Actinomycetota) *Bifidobacterium anseris*	0 ± 0	0 ± 0	0 ± 0	0 ± 0
(Actinomycetota) *Bifidobacterium apri*	0 ± 0	0 ± 0	0 ± 0	0 ± 0
(Actinomycetota) *Bifidobacterium biavatii*	0 ± 0	0,0006 ± 0,0036	0 ± 0	0 ± 0
(Actinomycetota) *Bifidobacterium bifidum*	1,5771 ± 3,3915	0,387 ± 0,7544	0,6031 ± 1,0969	0,4112 ± 0,9322
(Actinomycetota) *Bifidobacterium boum*	0 ± 0	0 ± 0	0 ± 0	0 ± 0
(Actinomycetota) *Bifidobacterium breve*	0,014 ± 0,0432	0,0333 ± 0,1008	0,0121 ± 0,0313	0,0024 ± 0,0069

(Actinomycetota) *Bifidobacterium catenulatum*	0,3056 ± 1,456	0,0513 ± 0,1863	0,0006 ± 0,0036	0,0746 ± 0,3351
(Actinomycetota) *Bifidobacterium choerinum*	0 ± 0	0,0023 ± 0,0127	0 ± 0	0,0053 ± 0,0292
(Actinomycetota) *Bifidobacterium dentium*	0,0723 ± 0,2101	0,003 ± 0,0164	0 ± 0	0,0183 ± 0,1004
(Actinomycetota) *Bifidobacterium faecale*	0,0952 ± 0,5217	0,0023 ± 0,0062	0,044 ± 0,1762	0,18 ± 0,5629
(Actinomycetota) *Bifidobacterium gallicum*	0 ± 0	0,0023 ± 0,0089	0,0003 ± 0,0018	0 ± 0
(Actinomycetota) *Bifidobacterium italicum*	0 ± 0	0 ± 0	0 ± 0	0 ± 0
(Actinomycetota) *Bifidobacterium kashiwanohense*	0 ± 0	0 ± 0	0 ± 0	0 ± 0
(Actinomycetota) *Bifidobacterium longum*	3,9406 ± 5,1402	1,9686 ± 2,9118	1,7736 ± 1,7664	1,4329 ± 2,2837
(Actinomycetota) *Bifidobacterium merycicum*	0 ± 0	0 ± 0	0 ± 0	0 ± 0
(Actinomycetota) *Bifidobacterium moukalabense*	0 ± 0	0,0276 ± 0,1194	0 ± 0	0 ± 0
(Actinomycetota) *Bifidobacterium pseudocatenulatum*	0,3716 ± 1,4205	0,7236 ± 1,6512	0,6219 ± 1,3311	0,4988 ± 1,2014
(Actinomycetota) *Bifidobacterium pseudolongum*	0 ± 0	0,0083 ± 0,0282	0 ± 0	0,0016 ± 0,0074
(Actinomycetota) *Bifidobacterium pullorum*	0 ± 0	0 ± 0	0 ± 0	0 ± 0
(Actinomycetota) *Bifidobacterium reuteri*	0 ± 0	0,0013 ± 0,0073	0 ± 0	0 ± 0
(Actinomycetota) *Bifidobacterium ruminantium*	0 ± 0	0 ± 0	0 ± 0	0 ± 0
(Actinomycetota) *Bifidobacterium saguini*	0 ± 0	0,0093 ± 0,0313	0,0021 ± 0,0067	0,002 ± 0,0066
(Actinomycetota) *Bifidobacterium scardovii*	0 ± 0	0 ± 0	0 ± 0	0,001 ± 0,0054
(Actinomycetota) *Bifidobacterium stercoris*	0 ± 0	0 ± 0	0 ± 0	0 ± 0
(Actinomycetota) *Brevibacterium epidermidis*	0 ± 0	0 ± 0	0 ± 0	0 ± 0
(Actinomycetota) *Brevibacterium mcbrellneri*	0 ± 0	0 ± 0	0 ± 0	0 ± 0
(Actinomycetota) *Brevibacterium ravenspurgense*	0 ± 0	0 ± 0	0 ± 0	0 ± 0
(Actinomycetota) *Brevilactibacter sinopodophylli*	0 ± 0	0 ± 0	0 ± 0	0 ± 0
(Actinomycetota) *Cellulosimicrobium cellulans*	0 ± 0	0 ± 0	0 ± 0	0 ± 0
(Actinomycetota) *Collinsella aerofaciens*	3,8182 ± 3,8152	2,796 ± 3,1616	1,7479 ± 1,8107	2,0356 ± 2,7286
(Actinomycetota) *Collinsella bouchesdurhonensis*	0,0081 ± 0,0443	0,0023 ± 0,0127	0 ± 0	0 ± 0
(Actinomycetota) *Collinsella intestinalis*	0 ± 0	0 ± 0	0 ± 0	0 ± 0
(Actinomycetota) *Collinsella massiliensis*	0 ± 0	0 ± 0	0 ± 0	0 ± 0
(Actinomycetota) *Collinsella stercoris*	0 ± 0	0 ± 0	0 ± 0	0,0006 ± 0,0036
(Actinomycetota) *Collinsella Tanakaei*	0 ± 0	0 ± 0	0,0146 ± 0,0596	0,01 ± 0,0547
(Actinomycetota) *Corynebacterium amycolatum*	0,0013 ± 0,0073	0 ± 0	0 ± 0	0 ± 0
(Actinomycetota) *Corynebacterium argentoratense*	0,0016 ± 0,0091	0 ± 0	0 ± 0	0 ± 0
(Actinomycetota) *Corynebacterium aurimucosum*	0,0007 ± 0,0038	0,0033 ± 0,0182	0 ± 0	0 ± 0
(Actinomycetota) *Corynebacterium coyleae*	0 ± 0	0 ± 0	0 ± 0	0 ± 0
(Actinomycetota) *Corynebacterium durum*	0,002 ± 0,008	0,0016 ± 0,0074	0 ± 0	0 ± 0
(Actinomycetota) *Corynebacterium jeikeium*	0 ± 0	0 ± 0	0 ± 0	0 ± 0
(Actinomycetota) *Corynebacterium matruchotii*	0 ± 0	0 ± 0	0 ± 0	0 ± 0
(Actinomycetota) *Corynebacterium mucifaciens*	0 ± 0	0,0016 ± 0,0091	0 ± 0	0 ± 0

(Actinomycetota) *Corynebacterium pseudodiphtheriticum*	0 ± 0	0 ± 0	0 ± 0	0 ± 0
(Actinomycetota) *Corynebacterium pyruviciproducens*	0 ± 0	0 ± 0	0 ± 0	0 ± 0
(Actinomycetota) *Corynebacterium simulans*	0 ± 0	0 ± 0	0 ± 0	0 ± 0
(Actinomycetota) *Corynebacterium sundsvallense*	0 ± 0	0,0066 ± 0,0365	0 ± 0	0 ± 0
(Actinomycetota) *Corynebacterium tuberculostearicum*	0 ± 0	0 ± 0	0 ± 0	0 ± 0
(Actinomycetota) *Cutibacterium acnes*	0 ± 0	0 ± 0	0 ± 0	0 ± 0
(Actinomycetota) *Denitrobacterium detoxificans*	0 ± 0	0 ± 0	0 ± 0	0 ± 0
(Actinomycetota) *Eggerthella lenta*	0,5896 ± 1,2144	0,421 ± 0,9106	0,2952 ± 0,9892	0,2232 ± 0,4554
(Actinomycetota) *Eggerthella sinensis*	0,0186 ± 0,0867	0,0016 ± 0,0091	0,001 ± 0,0054	0 ± 0
(Actinomycetota) *Eggerthella timonensis*	0,0091 ± 0,0498	0,547 ± 1,6873	0,0696 ± 0,2006	0,0323 ± 0,1029
(Actinomycetota) *Enorma massiliensis*	0 ± 0	0 ± 0	0 ± 0	0,0003 ± 0,0018
(Actinomycetota) *Enorma phocaeensis*	0 ± 0	0 ± 0	0 ± 0	0 ± 0
(Actinomycetota) *Enorma timonensis*	0 ± 0	0 ± 0	0 ± 0	0 ± 0
(Actinomycetota) *Enteroscipio rubneri*	0,0006 ± 0,0036	0 ± 0	0 ± 0	0 ± 0
(Actinomycetota) *Fannyhessea vaginae*	0 ± 0	0 ± 0	0 ± 0	0 ± 0
(Actinomycetota) *Flaviflexus huanghaiensis*	0 ± 0	0 ± 0	0 ± 0	0 ± 0
(Actinomycetota) *Gardnerella leopoldii*	0 ± 0	0 ± 0	0 ± 0	0 ± 0
(Actinomycetota) *Gardnerella swidsinskii*	0 ± 0	0 ± 0	0 ± 0	0 ± 0
(Actinomycetota) *Gordonibacter faecihominis*	0 ± 0	0 ± 0	0 ± 0	0 ± 0
(Actinomycetota) *Gordonibacter pamelaeae*	0,1333 ± 0,3826	0,042 ± 0,0844	0,0276 ± 0,0776	0,1137 ± 0,5191
(Actinomycetota) *Gordonibacter urolithinfaciens*	0,0236 ± 0,0823	0 ± 0	0,0003 ± 0,0018	0 ± 0
(Actinomycetota) *Lancefieldella rimae*	0,0006 ± 0,0036	0,0023 ± 0,0072	0,0003 ± 0,0018	0 ± 0
(Actinomycetota) *Lawsonella clevelandensis*	0,0046 ± 0,0255	0 ± 0	0 ± 0	0 ± 0
(Actinomycetota) *Leucobacter iarius*	0 ± 0	0 ± 0	0 ± 0	0 ± 0
(Actinomycetota) *Microbacterium phyllosphaerae*	0 ± 0	0 ± 0	0 ± 0	0 ± 0
(Actinomycetota) *Mobiluncus curtisii*	0,0023 ± 0,0127	0 ± 0	0 ± 0	0 ± 0
(Actinomycetota) *Mycobacterium porcinum*	0 ± 0	0,0003 ± 0,0018	0 ± 0	0 ± 0
(Actinomycetota) *Olsenella profusa*	0,0003 ± 0,0018	0,0003 ± 0,0018	0 ± 0	0 ± 0
(Actinomycetota) *Olsenella scatoligenes*	0 ± 0	0 ± 0	0 ± 0	0 ± 0
(Actinomycetota) *Olsenella uli*	0,0023 ± 0,0127	0,0243 ± 0,0644	0,0773 ± 0,4235	0,083 ± 0,4546
(Actinomycetota) *Paraeggerthella hongkongensis*	0 ± 0	0,001 ± 0,0054	0 ± 0	0 ± 0
(Actinomycetota) *Parascardovia denticolens*	0 ± 0	0 ± 0	0 ± 0	0 ± 0
(Actinomycetota) *Parolsenella catena*	0 ± 0	0,0063 ± 0,0346	0 ± 0	0 ± 0
(Actinomycetota) *Parolsenella massiliensis*	0 ± 0	0,015 ± 0,0565	0 ± 0	0 ± 0
(Actinomycetota) *Propionibacterium acidifaciens*	0 ± 0	0 ± 0	0 ± 0	0,0016 ± 0,0091
(Actinomycetota) *Propionibacterium freudenreichii*	0,008 ± 0,0438	0 ± 0	0 ± 0	0 ± 0
(Actinomycetota) *Raoultibacter massiliensis*	0 ± 0	0,0196 ± 0,0678	0,0006 ± 0,0025	0,001 ± 0,0054
(Actinomycetota) *Raoultibacter timonensis*	0,0217 ± 0,0503	0,0823 ± 0,2146	0,0287 ± 0,0663	0,0639 ± 0,1046

(Actinomycetota) *Rothia aeria*	0 ± 0	0 ± 0	0 ± 0	0 ± 0
(Actinomycetota) *Rothia dentocariosa*	0,0006 ± 0,0036	0,0003 ± 0,0018	0 ± 0	0 ± 0
(Actinomycetota) *Rothia mucilaginosa*	0,0006 ± 0,0036	0,0053 ± 0,0179	0,0003 ± 0,0018	0 ± 0
(Actinomycetota) *Rubneribacter badeniensis*	0 ± 0	0 ± 0	0 ± 0	0 ± 0
(Actinomycetota) *Scardovia wiggsiae*	0 ± 0	0,0046 ± 0,0255	0 ± 0	0 ± 0
(Actinomycetota) *Schaalia canis*	0 ± 0	0 ± 0	0 ± 0	0 ± 0
(Actinomycetota) *Schaalia cardiffensis*	0 ± 0	0 ± 0	0 ± 0	0 ± 0
(Actinomycetota) *Schaalia odontolytica*	0,0023 ± 0,0127	0,0533 ± 0,0804	0,0136 ± 0,0345	0,016 ± 0,0444
(Actinomycetota) *Schaalia turicensis*	0 ± 0	0,0006 ± 0,0036	0 ± 0	0 ± 0
(Actinomycetota) *Senegalimassilia anaerobia*	0,091 ± 0,1732	0,438 ± 1,6098	0,1685 ± 0,3044	0,1147 ± 0,2451
(Actinomycetota) *Slackia equolifaciens*	0 ± 0	0,0053 ± 0,0256	0 ± 0	0 ± 0
(Actinomycetota) *Slackia exigua*	0 ± 0	0 ± 0	0 ± 0	0 ± 0
(Actinomycetota) *Slackia faecicanis*	0 ± 0	0,021 ± 0,0711	0,0113 ± 0,062	0 ± 0
(Actinomycetota) *Slackia isoflavoniconvertens*	0,028 ± 0,0841	0,0813 ± 0,1471	0,0436 ± 0,089	0,032 ± 0,0882
(Actinomycetota) *Slackia piriformis*	0,02 ± 0,1022	0 ± 0	0,002 ± 0,0109	0 ± 0
(Actinomycetota) *Sporichthya polymorpha*	0 ± 0	0 ± 0	0 ± 0	0 ± 0
(Actinomycetota) *Streptomyces gramineus*	0 ± 0	0 ± 0	0 ± 0	0 ± 0
(Actinomycetota) *Streptomyces tendae*	0 ± 0	0 ± 0	0 ± 0	0 ± 0
(Actinomycetota) *Thalassiella azotivora*	0 ± 0	0 ± 0	0 ± 0	0 ± 0
(Actinomycetota) *Trueperella abortisuis*	0 ± 0	0 ± 0	0 ± 0	0,0003 ± 0,0018
(Actinomycetota) *Trueperella pyogenes*	0 ± 0	0 ± 0	0 ± 0	0 ± 0
(Actinomycetota) *Varibaculum cambriense*	0,0033 ± 0,0182	0 ± 0	0,0006 ± 0,0036	0 ± 0
(Bacteroidota) *Prevotella disiens*	0 ± 0	0 ± 0	0 ± 0	0 ± 0
(Bacteroidota) *Alistipes Alistipes*	0 ± 0	0 ± 0	0 ± 0	0 ± 0
(Bacteroidota) *Alistipes finegoldii*	0,2463 ± 0,6755	0,02 ± 0,0685	0,4617 ± 1,1887	0,1936 ± 0,5383
(Bacteroidota) *Alistipes ihumii*	0,0059 ± 0,0324	0,0373 ± 0,1158	0,0223 ± 0,0537	0,0346 ± 0,1109
(Bacteroidota) *Alistipes indistinctus*	0,0126 ± 0,0491	0,045 ± 0,1172	0,0303 ± 0,0589	0,0805 ± 0,1943
(Bacteroidota) *Alistipes inops*	0 ± 0	0 ± 0	0,0083 ± 0,0456	0,0046 ± 0,0255
(Bacteroidota) *Alistipes massiliensis*	0 ± 0	0 ± 0	0 ± 0	0 ± 0
(Bacteroidota) *Alistipes obesi*	0,0473 ± 0,1455	0 ± 0	0,0693 ± 0,2304	0,139 ± 0,5349
(Bacteroidota) *Alistipes onderdonkii*	0,0657 ± 0,3579	0,3166 ± 0,9555	0,226 ± 0,4765	0,1314 ± 0,2773
(Bacteroidota) *Alistipes putredinis*	0,568 ± 0,6485	0,5256 ± 0,6043	1,0211 ± 1,187	0,9796 ± 1,054
(Bacteroidota) *Alistipes senegalensis*	0 ± 0	0,007 ± 0,0205	0 ± 0	0 ± 0
(Bacteroidota) *Alistipes shahii*	0,0529 ± 0,1375	0,1723 ± 0,2461	0,1262 ± 0,2198	0,2008 ± 0,3863
(Bacteroidota) *Alistipes timonensis*	0 ± 0	0 ± 0	0 ± 0	0,0043 ± 0,0237
(Bacteroidota) *Alloprevotella rava*	0,0133 ± 0,073	0,0133 ± 0,073	0 ± 0	0 ± 0
(Bacteroidota) *Anaerophaga thermohalophila*	0 ± 0	0 ± 0	0 ± 0	0 ± 0
(Bacteroidota) *Bacteriodes koreensis*	0,0145 ± 0,0794	0,004 ± 0,0219	0 ± 0	0,032 ± 0,1422

(Bacteroidota) *Bacteroides acidifaciens*	0 ± 0	0 ± 0	0 ± 0	0 ± 0
(Bacteroidota) *Bacteroides barnesiae*	0 ± 0	0 ± 0	0 ± 0	0 ± 0
(Bacteroidota) *Bacteroides caccae*	0,7793 ± 1,9786	0,4193 ± 0,6482	0,6894 ± 1,2589	0,398 ± 0,6592
(Bacteroidota) *Bacteroides caecigallinarum*	0 ± 0	0 ± 0	0 ± 0	0 ± 0
(Bacteroidota) *Bacteroides caecimuris*	0,0041 ± 0,0226	0,0006 ± 0,0036	0,0013 ± 0,0073	0,0063 ± 0,0346
(Bacteroidota) *Bacteroides cellulosilyticus*	0,0113 ± 0,0584	0,084 ± 0,2937	0,0745 ± 0,2496	0,1855 ± 0,5829
(Bacteroidota) *Bacteroides clarus*	0 ± 0	0,0263 ± 0,0868	0,025 ± 0,0658	0,125 ± 0,3881
(Bacteroidota) *Bacteroides coprocola*	0 ± 0	0 ± 0	0,1473 ± 0,5507	0,1326 ± 0,7061
(Bacteroidota) *Phocaeicola coprophilus*	0,0043 ± 0,0202	0 ± 0	0 ± 0	0 ± 0
(Bacteroidota) *Phocaeicola dorei*	0,5433 ± 2,0454	0 ± 0	0,8556 ± 2,9405	0,3196 ± 0,9209
(Bacteroidota) *Bacteroides eggerthii*	0,117 ± 0,5111	0,1133 ± 0,2991	0,594 ± 1,3981	0,3551 ± 1,2509
(Bacteroidota) *Bacteroides faecichinchillae*	0 ± 0	0 ± 0	0 ± 0	0 ± 0
(Bacteroidota) *Bacteroides faecis*	0,0683 ± 0,249	0,1233 ± 0,2476	0,1509 ± 0,3095	0,4898 ± 1,4
(Bacteroidota) *Bacteroides finegoldii*	0 ± 0	0,0183 ± 0,0724	0,1109 ± 0,323	0,0884 ± 0,2526
(Bacteroidota) *Bacteroides fluxus*	0,023 ± 0,1259	0 ± 0	0,0986 ± 0,5385	0,003 ± 0,0164
(Bacteroidota) *Bacteroides fragilis*	0,4627 ± 2,0734	0,324 ± 0,9291	0,2177 ± 0,9855	0,2404 ± 0,8353
(Bacteroidota) *Bacteroides galacturonicus*	0,011 ± 0,0602	0 ± 0	0,0296 ± 0,1299	0,019 ± 0,0773
(Bacteroidota) *Bacteroides gallinaceum*	0 ± 0	0,069 ± 0,3779	0 ± 0	0 ± 0
(Bacteroidota) *Bacteroides gallinarum*	0 ± 0	0 ± 0	0 ± 0	0 ± 0
(Bacteroidota) *Bacteroides intestinalis*	0 ± 0	0 ± 0	0,0003 ± 0,0018	0,0586 ± 0,1803
(Bacteroidota) *Bacteroides koreensis*	0,0076 ± 0,0419	0,009 ± 0,0423	0,001 ± 0,0054	0,025 ± 0,1369
(Bacteroidota) *Bacteroides kribbi*	0 ± 0	0 ± 0	0,0714 ± 0,2688	0 ± 0
(Bacteroidota) *Bacteroides massiliensis*	0,0203 ± 0,0537	0 ± 0	0,1523 ± 0,4278	0,1503 ± 0,6188
(Bacteroidota) *Bacteroides mediterraneensis*	0,0733 ± 0,4018	0,1513 ± 0,6646	0,1856 ± 1,0169	0,2373 ± 1,2224
(Bacteroidota) *Bacteroides nordii*	0,0296 ± 0,1624	0,1036 ± 0,5396	0,0056 ± 0,031	0,0186 ± 0,0666
(Bacteroidota) *Bacteroides oleiciplenus*	0 ± 0	0 ± 0	0 ± 0	0 ± 0
(Bacteroidota) *Bacteroides ovatus*	0,1216 ± 0,2735	0,6483 ± 2,1358	0,8147 ± 2,3916	0,5272 ± 0,7774
(Bacteroidota) *Phocaeicola plebeius*	0,0956 ± 0,3378	0 ± 0	1,0816 ± 3,1557	0,457 ± 1,5923
(Bacteroidota) *Bacteroides rodentium*	0 ± 0	0,012 ± 0,0209	0,0227 ± 0,0763	0,0183 ± 0,0619
(Bacteroidota) *Bacteroides salyersiae*	0,0263 ± 0,1143	0,0366 ± 0,1326	0,0334 ± 0,1037	0,0113 ± 0,0355
(Bacteroidota) *Bacteroides stercorirosoris*	0 ± 0	0 ± 0	0 ± 0	0 ± 0
(Bacteroidota) *Bacteroides stercoris*	0,5712 ± 1,4794	0,585 ± 0,9326	1,2031 ± 2,832	0,7643 ± 2,3374
(Bacteroidota) *Bacteroides thetaiotaomicron*	0,1272 ± 0,3235	0,1496 ± 0,2975	0,6333 ± 2,004	0,286 ± 0,6193
(Bacteroidota) *Bacteroides timonensis*	0 ± 0	0,002 ± 0,0109	0 ± 0	0 ± 0
(Bacteroidota) *Bacteroides uniformis*	1,2209 ± 1,4826	1,1273 ± 1,435	2,9766 ± 3,3074	4,6345 ± 10,3839
(Bacteroidota) *Phocaeicola vulgatus*	0,5613 ± 0,9865	0,0576 ± 0,3158	1,5926 ± 3,5094	1,9603 ± 4,5647
(Bacteroidota) *Bacteroides xylanisolvens*	0,2499 ± 0,7561	0,3313 ± 0,5081	0,3624 ± 0,689	0,5498 ± 1,0187
(Bacteroidota) *Barnesiella intestinihominis*	0,3109 ± 0,4538	0,3766 ± 0,4543	0,6418 ± 0,7758	0,671 ± 1,1149

(Bacteroidota) *Butyricimonas faecihominis*	0,0438 ± 0,1354	0,0213 ± 0,061	0,019 ± 0,0549	0,0115 ± 0,0455
(Bacteroidota) *Butyricimonas paravirosa*	0,0068 ± 0,0374	0,0003 ± 0,0018	0,0006 ± 0,0036	0,0006 ± 0,0036
(Bacteroidota) *Butyricimonas phoceensis*	0,0016 ± 0,0091	0,048 ± 0,1874	0,013 ± 0,0642	0,0036 ± 0,0167
(Bacteroidota) *Butyricimonas synergistica*	0,0016 ± 0,0091	0 ± 0	0,0159 ± 0,0509	0,0174 ± 0,0487
(Bacteroidota) *Butyricimonas virosa*	0,007 ± 0,0258	0,0393 ± 0,1024	0,0264 ± 0,061	0,1914 ± 0,4834
(Bacteroidota) *Coprobacter fastidiosus*	0,028 ± 0,1415	0,0176 ± 0,0414	0,0278 ± 0,0878	0,0512 ± 0,1608
(Bacteroidota) *Coprobacter fastidious*	0,0027 ± 0,0147	0 ± 0	0,0053 ± 0,0292	0,0006 ± 0,0036
(Bacteroidota) *Coprobacter secundus*	0 ± 0	0 ± 0	0 ± 0	0 ± 0
(Bacteroidota) *Crocinitomix catalasitica*	0 ± 0	0 ± 0	0 ± 0	0 ± 0
(Bacteroidota) *Culturomica massiliensis*	0 ± 0	0,07 ± 0,3796	0 ± 0	0 ± 0
(Bacteroidota) *Duncaniella freteri*	0,006 ± 0,0328	0,0076 ± 0,033	0,0521 ± 0,2116	0,0885 ± 0,2343
(Bacteroidota) *Dysgonomonas capnocytophagoides*	0 ± 0	0 ± 0	0 ± 0	0 ± 0
(Bacteroidota) *Dysgonomonas mossii*	0 ± 0	0 ± 0	0 ± 0	0 ± 0
(Bacteroidota) *Dysgonomonas oryzarvi*	0 ± 0	0 ± 0	0 ± 0	0 ± 0
(Bacteroidota) *Edaphobaculum flavum*	0 ± 0	0,1726 ± 0,6418	0 ± 0	0,0113 ± 0,062
(Bacteroidota) *Eudoraea chungangensis*	0,0048 ± 0,0266	0,007 ± 0,0216	0 ± 0	0 ± 0
(Bacteroidota) *Flavobacterium urocaniciphilum*	0 ± 0	0,0043 ± 0,0237	0 ± 0	0 ± 0
(Bacteroidota) *Gabonia massiliensis*	0,0013 ± 0,0073	0,0606 ± 0,3118	0,0121 ± 0,0371	0,0102 ± 0,0354
(Bacteroidota) *Gabonibacter massiliensis*	0 ± 0	0,0123 ± 0,0534	0,0013 ± 0,0074	0 ± 0
(Bacteroidota) *Galbibacter mesophilus*	0 ± 0	0 ± 0	0 ± 0	0 ± 0
(Bacteroidota) *Ihubacter massiliensis*	0,0067 ± 0,0227	0,0936 ± 0,0929	0,0443 ± 0,1397	0,0392 ± 0,145
(Bacteroidota) *Lentimicrobium saccharophilu*	0 ± 0	0 ± 0	0 ± 0	0 ± 0
(Bacteroidota) *Lishizhenia tianjinensis*	0 ± 0	0 ± 0	0 ± 0	0 ± 0
(Bacteroidota) *Marinilabilia salmonicolor*	0 ± 0	0 ± 0	0,043 ± 0,2355	0,0386 ± 0,2117
(Bacteroidota) *Marseilla massiliensis*	0 ± 0	0,02 ± 0,1095	0,027 ± 0,1478	0,0356 ± 0,1953
(Bacteroidota) *Metaprevotella massiliensis*	0 ± 0	0 ± 0	0 ± 0	0 ± 0
(Bacteroidota) *Microscilla marina*	0 ± 0	0 ± 0	0 ± 0	0 ± 0
(Bacteroidota) *Muribaculum intestinale*	0 ± 0	0,1573 ± 0,3628	0,146 ± 0,5559	0,0954 ± 0,3198
(Bacteroidota) *Odoribacter laneus*	0 ± 0	0,0006 ± 0,0036	0,007 ± 0,0316	0,0116 ± 0,0639
(Bacteroidota) *Odoribacter splanchnicus*	0,0911 ± 0,1415	0,1166 ± 0,2052	0,278 ± 0,3405	0,2093 ± 0,2977
(Bacteroidota) *Paludibacter propionicigenes*	0 ± 0	0 ± 0	0 ± 0	0 ± 0
(Bacteroidota) *Parabacteroides chongii*	0,0019 ± 0,0107	0,003 ± 0,0131	0,0106 ± 0,0413	0 ± 0
(Bacteroidota) *Parabacteroides distasonis*	0,3203 ± 0,6423	0,3186 ± 0,9598	0,7338 ± 0,7976	0,7737 ± 1,1091
(Bacteroidota) *Parabacteroides faecis*	0 ± 0	0 ± 0	0 ± 0	0 ± 0
(Bacteroidota) *Parabacteroides goldsteinii*	0,0083 ± 0,0456	0,0036 ± 0,0154	0,0227 ± 0,1243	0,041 ± 0,1508
(Bacteroidota) *Parabacteroides gordonii*	0 ± 0	0 ± 0	0 ± 0	0,0003 ± 0,0018
(Bacteroidota) *Parabacteroides johnsonii*	0 ± 0	0,0013 ± 0,0073	0,0551 ± 0,1853	0,1019 ± 0,31
(Bacteroidota) *Parabacteroides merdae*	0,2009 ± 0,4048	0,5463 ± 1,2623	0,3554 ± 0,4636	0,3372 ± 0,6187

(Bacteroidota) *Parapedobacter soli*	0 ± 0	0 ± 0	0 ± 0	0 ± 0
(Bacteroidota) *Paraprevotella clara*	0,0463 ± 0,2145	0,16 ± 0,3275	0,1247 ± 0,2387	0,3241 ± 0,7677
(Bacteroidota) *Paraprevotella xylaniphila*	0 ± 0	0 ± 0	0,0046 ± 0,0255	0,002 ± 0,008
(Bacteroidota) *Pedobacter jejuensis*	0 ± 0	0,002 ± 0,0109	0 ± 0	0,0405 ± 0,2221
(Bacteroidota*) Pedobacter quisquiliarum*	0 ± 0	0 ± 0	0 ± 0	0 ± 0
(Bacteroidota) *Pedobacter terrae*	0 ± 0	0 ± 0	0 ± 0	0 ± 0
(Bacteroidota) *Phocaeicola barnesiae*	0 ± 0	0,1893 ± 1,037	0 ± 0	0 ± 0
(Bacteroidota) *Phocaeicola coprocola*	0,3311 ± 1,2618	0,6146 ± 1,9248	0,47 ± 1,1858	0,5439 ± 1,3868
(Bacteroidota) *Phocaeicola coprophilus*	0 ± 0	0,0993 ± 0,342	0,4753 ± 2,6036	0,0006 ± 0,0034
(Bacteroidota) *Phocaeicola dorei*	0,2248 ± 0,7727	0,488 ± 1,2703	1,051 ± 2,5945	1,0207 ± 1,7763
(Bacteroidota) *Phocaeicola massiliensis*	0 ± 0	0,2936 ± 0,6673	0,8135 ± 1,3197	0,9579 ± 1,8306
(Bacteroidota) *Phocaeicola plebeius*	0 ± 0	0,1576 ± 0,4018	0,1303 ± 0,5501	0,7042 ± 1,9619
(Bacteroidota) *Phocaeicola salanitronis*	0,003 ± 0,0164	0,015 ± 0,0645	0,0163 ± 0,0638	0,0366 ± 0,2008
(Bacteroidota) *Phocaeicola sartorii*	0 ± 0	0,053 ± 0,2902	0,0012 ± 0,0065	0,0006 ± 0,0036
(Bacteroidota) *Phocaeicola vulgatus*	0,7073 ± 1,994	1,8343 ± 2,4126	3,5035 ± 6,3278	2,471 ± 3,5455
(Bacteroidota) *Porphyromonas asaccharolytica*	0,021 ± 0,1025	0 ± 0	0,0023 ± 0,0127	0 ± 0
(Bacteroidota) *Porphyromonas bennonis*	0 ± 0	0 ± 0	0 ± 0	0 ± 0
(Bacteroidota) *Porphyromonas circumdentaria*	0 ± 0	0 ± 0	0 ± 0	0 ± 0
(Bacteroidota) *Porphyromonas gingivalis*	0,002 ± 0,0109	0 ± 0	0 ± 0	0 ± 0
(Bacteroidota) *Porphyromonas somerae*	0 ± 0	0 ± 0	0 ± 0	0 ± 0
(Bacteroidota) *Porphyromonas uenonis*	0 ± 0	0 ± 0	0 ± 0	0 ± 0
(Bacteroidota) *Prevotella amnii*	0 ± 0	0,0306 ± 0,1679	0 ± 0	0 ± 0
(Bacteroidota) *Prevotella bergensis*	0,0016 ± 0,0091	0 ± 0	0 ± 0	0 ± 0
(Bacteroidota) *Prevotella bivia*	0 ± 0	0 ± 0	0,0559 ± 0,1983	0 ± 0
(Bacteroidota) *Prevotella buccae*	0 ± 0	0 ± 0	0 ± 0	0 ± 0
(Bacteroidota) *Prevotella buccalis*	0,1023 ± 0,5605	0 ± 0	0 ± 0	0 ± 0
(Bacteroidota) *Prevotella colorans*	0 ± 0	0,0006 ± 0,0036	0 ± 0	0 ± 0
(Bacteroidota) *Prevotella copri*	2,1452 ± 6,5542	3,9736 ± 6,9649	4,7111 ± 11,3411	3,4468 ± 7,9155
(Bacteroidota) *Prevotella corporis*	0,001 ± 0,0054	0 ± 0	0,005 ± 0,019	0 ± 0
(Bacteroidota) *Prevotella dentalis*	0 ± 0	0 ± 0	0 ± 0	0 ± 0
(Bacteroidota) *Prevotella denticola*	0 ± 0	0,0493 ± 0,1775	0,0016 ± 0,0091	0,0036 ± 0,02
(Bacteroidota) *Prevotella disiens*	0,014 ± 0,0766	0 ± 0	0 ± 0	0,0006 ± 0,0036
(Bacteroidota) *Prevotella histicola*	0 ± 0	0 ± 0	0 ± 0	0 ± 0
(Bacteroidota) *Prevotella marshii*	0 ± 0	0 ± 0	0 ± 0	0,0233 ± 0,1279
(Bacteroidota) *Prevotella melaninogenica*	0 ± 0	0 ± 0	0 ± 0	0 ± 0
(Bacteroidota) *Prevotella multisaccharivorax*	0 ± 0	0,005 ± 0,0273	0 ± 0	0 ± 0
(Bacteroidota) *Prevotella oralis*	0 ± 0	0 ± 0	0 ± 0	0 ± 0
(Bacteroidota) *Prevotella oris*	0 ± 0	0 ± 0	0 ± 0	0 ± 0

(Bacteroidota) *Prevotella oryzae*	0,0026 ± 0,0146	0,0646 ± 0,2141	0,4106 ± 1,9857	0,0023 ± 0,0127
(Bacteroidota) *Prevotella oulorum*	0 ± 0	0,006 ± 0,0328	0 ± 0	0 ± 0
(Bacteroidota) *Prevotella saccharolytica*	0,0036 ± 0,0182	0,1353 ± 0,5275	0 ± 0	0 ± 0
(Bacteroidota) *Prevotella salivae*	0,105 ± 0,5751	0,0126 ± 0,0675	0 ± 0	0 ± 0
(Bacteroidota) *Prevotella scopos*	0 ± 0	0,0063 ± 0,028	0 ± 0	0 ± 0
(Bacteroidota) *Prevotella shahii*	0 ± 0	0,0026 ± 0,0146	0,008 ± 0,0438	0,0023 ± 0,0127
(Bacteroidota) *Prevotella stercorea*	0,0166 ± 0,0912	0,2833 ± 1,1033	0,0153 ± 0,0839	0,0023 ± 0,0097
(Bacteroidota) *Prevotella timonensis*	0 ± 0	0 ± 0	0,0038 ± 0,0208	0,005 ± 0,0273
(Bacteroidota) *Prevotella veroralis*	0 ± 0	0 ± 0	0 ± 0	0 ± 0
(Bacteroidota) *Prevotellamassilia timonensis*	0 ± 0	0,2696 ± 0,8611	0,0503 ± 0,2756	0,0673 ± 0,3687
(Bacteroidota) *Roseimarinus sediminis*	0 ± 0	0,072 ± 0,2333	0,0306 ± 0,1679	0,0462 ± 0,2205
(Bacteroidota) *Rufibacter quisquiliarum*	0 ± 0	0,0006 ± 0,0025	0 ± 0	0 ± 0
(Bacteroidota) *Sanguibacteroides justesenii*	0,0003 ± 0,0018	0 ± 0	0 ± 0	0 ± 0
(Bacteroidota) *Sunxiuqinia faeciviva*	0 ± 0	0 ± 0	0 ± 0	0 ± 0
(Bacteroidota) *Tidjanibacter massiliensis*	0,0029 ± 0,0162	0,0133 ± 0,0431	0 ± 0	0,064 ± 0,2448
(Bacteroidota) *Zhouia amylolytica*	0,0005 ± 0,0029	0 ± 0	0 ± 0	0,0014 ± 0,008
(Bacteroidota) *Mediterranea massiliensis*	0,0467 ± 0,2559	0,033 ± 0,1265	0 ± 0	0 ± 0
(Cyanobacteriota) *Gloeobacter violaceus*	0 ± 0	0 ± 0	0 ± 0	0 ± 0
(Bacillota) *Eubacterium ventriosum*	0,0888 ± 0,2404	0,0913 ± 0,1145	0,0639 ± 0,0832	0,1475 ± 0,3767
(Bacillota) *Absiella tortuosum*	0 ± 0	0 ± 0	0,0006 ± 0,0036	0,0006 ± 0,0036
(Bacillota) *Abyssivirga alkaniphila*	0 ± 0	0 ± 0	0,0003 ± 0,0018	0 ± 0
(Bacillota) *Acetanaerobacterium elongatum*	0 ± 0	0,0033 ± 0,0102	0,001 ± 0,0054	0,001 ± 0,004
(Bacillota) *Acetivibrio aldrichii*	0 ± 0	0 ± 0	0 ± 0	0 ± 0
(Bacillota) *Acetivibrio cellulolyticus*	0 ± 0	0,0046 ± 0,0169	0 ± 0	0,0637 ± 0,3488
(Bacillota) *Acetivibrio clariflavus*	0,0073 ± 0,0401	0,0053 ± 0,0208	0,001 ± 0,0054	0,0086 ± 0,0474
(Bacillota) *Acetivibrio straminisolvens*	0,0683 ± 0,3742	0,0006 ± 0,0025	0 ± 0	0,108 ± 0,4107
(Bacillota) *Acetivibrio thermocellus*	0 ± 0	0,0023 ± 0,0097	0 ± 0	0 ± 0
(Bacillota) Acidaminobacter hydrogenoformans	0 ± 0	0 ± 0	0 ± 0	0 ± 0
(Bacillota) Acidaminococcus fermentans	0 ± 0	0,0033 ± 0,0129	0,0426 ± 0,1554	0,0726 ± 0,2905
(Bacillota) *Acidaminococcus intestini*	0,0036 ± 0,02	0,1113 ± 0,4249	0,1546 ± 0,5593	0,1539 ± 0,608
(Bacillota) *Acidaminococcus timonensis*	0,0006 ± 0,0036	0 ± 0	0 ± 0	0 ± 0
(Bacillota) *Acutalibacter muris*	0,0418 ± 0,2033	0,0296 ± 0,0576	0,0268 ± 0,0646	0,0064 ± 0,0177
(Bacillota) *Aerococcus sanguinicola*	0 ± 0	0 ± 0	0 ± 0	0 ± 0
(Bacillota) *Aerococcus suis*	0 ± 0	0,0016 ± 0,0059	0,0003 ± 0,0018	0 ± 0
(Bacillota) *Aerococcus urinae*	0 ± 0	0 ± 0	0 ± 0	0 ± 0
(Bacillota) *Agathobacter ruminis*	0 ± 0	0 ± 0	0 ± 0	0 ± 0
(Bacillota) *Agathobaculum butyriciproducens*	0,081 ± 0,2108	0,0696 ± 0,291	0,0116 ± 0,0407	0,023 ± 0,0874
(Bacillota) *Agathobaculum desmolans*	0 ± 0	0,0066 ± 0,0168	0,0013 ± 0,005	0,0151 ± 0,0774

(Bacillota) *Alkalibacter saccharofermentans*	0 ± 0	0 ± 0	0 ± 0	0 ± 0
(Bacillota) *Allisonella histaminiformans*	0,0013 ± 0,0073	0,0046 ± 0,0119	0,001 ± 0,0054	0,023 ± 0,0936
(Bacillota) *Alterileibacterium massiliense*	0 ± 0	0 ± 0	0 ± 0	0 ± 0
(Bacillota) *Amedibacillus dolichus*	0 ± 0	0,024 ± 0,0958	0,007 ± 0,0347	0,0368 ± 0,1219
(Bacillota) *Aminipila butyrica*	0,0169 ± 0,0724	0,118 ± 0,2563	0,0386 ± 0,0989	0,0312 ± 0,0841
(Bacillota) *Anaerobium acetethylicum*	0 ± 0	0,0013 ± 0,0073	0 ± 0	0 ± 0
(Bacillota) *Anaerobutyricum hallii*	0,2176 ± 0,5175	2,7543 ± 2,4665	1,8397 ± 2,2077	1,8281 ± 1,7794
(Bacillota) *Anaerococcus hydrogenalis*	0 ± 0	0 ± 0	0 ± 0	0 ± 0
(Bacillota) *Anaerococcus lactolyticus*	0 ± 0	0 ± 0	0 ± 0	0 ± 0
(Bacillota) *Anaerococcus obesiensis*	0 ± 0	0 ± 0	0 ± 0	0 ± 0
(Bacillota) *Anaerococcus octavius*	0 ± 0	0 ± 0	0 ± 0	0 ± 0
(Bacillota) *Anaerococcus prevotii*	0,0036 ± 0,02	0 ± 0	0 ± 0	0 ± 0
(Bacillota) *Anaerococcus urinomassiliensis*	0 ± 0	0 ± 0	0 ± 0	0 ± 0
(Bacillota) *Anaerococcus vaginalis*	0 ± 0	0 ± 0	0,0006 ± 0,0036	0 ± 0
(Bacillota) *Anaerocolumna aminovalerica*	0 ± 0	0 ± 0	0 ± 0	0 ± 0
(Bacillota) *Anaerocolumna cellulosilytica*	0,0961 ± 0,3637	0,0833 ± 0,2222	0,0835 ± 0,2063	0,2985 ± 1,0454
(Bacillota) *Anaerofilum pentosovorans*	0 ± 0	0,001 ± 0,004	0,001 ± 0,004	0,0009 ± 0,0052
(Bacillota) *Anaerofustis stercorihominis*	0 ± 0	0,0013 ± 0,0057	0 ± 0	0,001 ± 0,0054
(Bacillota) *Anaeroglobus geminatus*	0 ± 0	0 ± 0	0 ± 0	0 ± 0
(Bacillota) *Anaeromassilibacillus senegalensis*	0,0626 ± 0,206	0,119 ± 0,1173	0,139 ± 0,154	0,1542 ± 0,3503
(Bacillota) *Anaerostipes butyraticus*	0,0193 ± 0,1058	0,22 ± 1,1222	0 ± 0	0,0053 ± 0,019
(Bacillota) *Anaerostipes caccae*	0,003 ± 0,0164	0,011 ± 0,0463	0,0426 ± 0,1977	0,0126 ± 0,0657
(Bacillota) *Anaerostipes hadrus*	1,4463 ± 1,6659	2,65 ± 2,5756	2,009 ± 1,6723	2,0984 ± 2,1578
(Bacillota) *Anaerotaenia torta*	0,0023 ± 0,0127	0,0033 ± 0,0149	0,0026 ± 0,0101	0,0003 ± 0,0018
(Bacillota) *Anaerotignum aminivorans*	0 ± 0	0,01 ± 0,0194	0,0069 ± 0,0208	0,001 ± 0,0054
(Bacillota) *Anaerotignum faecicola*	0,0225 ± 0,0899	0,051 ± 0,0903	0,0314 ± 0,1066	0,0267 ± 0,0653
(Bacillota) *Anaerotignum lactatifermentans*	0 ± 0	0,0053 ± 0,0292	0,014 ± 0,0555	0,0293 ± 0,1606
(Bacillota) *Anaerotignum propionicum*	0 ± 0	0 ± 0	0 ± 0	0 ± 0
(Bacillota) *Anaerotruncus colihominis*	0,0053 ± 0,0256	0,0163 ± 0,0299	0,026 ± 0,1259	0,0097 ± 0,0266
(Bacillota) *Anaerotruncus rubiinfantis*	0,0015 ± 0,0061	0,045 ± 0,1111	0,0906 ± 0,4002	0,0711 ± 0,1805
(Bacillota) *Anaerovibrio lipolyticus*	0 ± 0	0,0413 ± 0,2189	0,2916 ± 1,5973	0 ± 0
(Bacillota) *Anaerovorax odorimutans*	0 ± 0	0,002 ± 0,0061	0 ± 0	0 ± 0
(Bacillota) *Angelakisella massiliensis*	0 ± 0	0 ± 0	0 ± 0	0 ± 0
(Bacillota) *Asaccharospora irregularis*	0 ± 0	0,0006 ± 0,0036	0 ± 0	0,0016 ± 0,0091
(Bacillota) *Bacillus horti*	0 ± 0	0 ± 0	0 ± 0	0 ± 0
(Bacillota) *Bacillus nealsonii*	0 ± 0	0 ± 0	0 ± 0	0,4168 ± 1,6731
(Bacillota) *Bacillus proteolyticus*	0 ± 0	0,0003 ± 0,0018	0 ± 0	0 ± 0
(Bacillota) *Bacillus subtilis*	0 ± 0	0 ± 0	0 ± 0	0 ± 0

(Bacillota) *Bacillus tropicus*	0 ± 0	0 ± 0	0 ± 0	0 ± 0
(Bacillota) *Bacteroides pectinophilus*	0,0153 ± 0,0839	0 ± 0	0,0207 ± 0,1133	0 ± 0
(Bacillota) *Bariatricus massiliensis*	0,0696 ± 0,3815	0,2156 ± 0,7714	0,2337 ± 0,5264	0,239 ± 0,6298
(Bacillota) *Beduini massiliensi*	0 ± 0	0 ± 0	0 ± 0	0,001 ± 0,0054
(Bacillota) *Bittarella massiliensis*	0 ± 0	0 ± 0	0 ± 0	0 ± 0
(Bacillota) *Blautia argi*	0,0726 ± 0,2768	0,2463 ± 0,4453	0,1251 ± 0,4309	0,292 ± 0,745
(Bacillota) *Blautia caecimuris*	0 ± 0	0,059 ± 0,2394	0,0113 ± 0,062	0,1395 ± 0,5847
(Bacillota) *Blautia coccoides*	0 ± 0	0,1183 ± 0,2874	0,0288 ± 0,1241	0,2708 ± 0,8623
(Bacillota) *Blautia faecicola*	0,0351 ± 0,1261	0,5233 ± 0,8648	0,345 ± 0,7238	0,4934 ± 0,867
(Bacillota) *Blautia faecis*	0,03 ± 0,1155	0,0776 ± 0,2172	0,0502 ± 0,1767	0,1382 ± 0,5888
(Bacillota) *Blautia glucerasea*	0,236 ± 0,5743	2,249 ± 2,2846	1,7531 ± 2,8677	1,46 ± 2,1226
(Bacillota) *Blautia hansenii*	0,0026 ± 0,0103	0,075 ± 0,1676	0,0101 ± 0,0294	0,0087 ± 0,0236
(Bacillota) *Blautia hominis*	0,002 ± 0,0109	0,0046 ± 0,022	0,0003 ± 0,0018	0,0056 ± 0,031
(Bacillota) *Blautia hydrogenotrophica*	0,0243 ± 0,075	0,051 ± 0,177	0,0046 ± 0,0173	0,0123 ± 0,0467
(Bacillota) *Blautia luti*	0,6157 ± 1,2666	5,6243 ± 4,3254	4,925 ± 5,136	4,959 ± 5,7875
(Bacillota) *Blautia obeum*	2,2803 ± 2,3148	0,5566 ± 0,7914	0,6384 ± 0,8776	1,4806 ± 2,5372
(Bacillota) *Blautia producta*	0,0496 ± 0,2682	0,0283 ± 0,0843	0,0226 ± 0,0615	0,008 ± 0,021
(Bacillota) *Blautia schinkii*	0,0003 ± 0,0018	0,0353 ± 0,0829	0,0193 ± 0,0637	0,0046 ± 0,0185
(Bacillota) *Blautia stercoris*	0,0253 ± 0,1387	0,132 ± 0,4666	0,0344 ± 0,1173	0,0682 ± 0,1715
(Bacillota) *Blautia torques*	0 ± 0	0 ± 0	0 ± 0	0 ± 0
(Bacillota) *Blautia wexlerae*	0,4315 ± 1,4137	0,4466 ± 0,7848	0,277 ± 0,6895	0,2276 ± 0,3667
(Bacillota) *Breznakia pachnodae*	0 ± 0	0,0056 ± 0,0185	0,001 ± 0,0054	0,0182 ± 0,0754
(Bacillota) *Bulleidia extructa*	0 ± 0	0,031 ± 0,1697	0 ± 0	0 ± 0
(Bacillota) *Butyribacterium methylotrophicum*	0 ± 0	0 ± 0	0 ± 0	0 ± 0
(Bacillota) *Butyricicoccus desmolans*	0 ± 0	0 ± 0	0 ± 0	0 ± 0
(Bacillota) *Butyricicoccus faecihominis*	0,127 ± 0,3697	0,415 ± 0,3986	0,3235 ± 0,4042	0,3709 ± 0,4638
(Bacillota) *Butyricicoccus pullicaecorum*	0 ± 0	0,0003 ± 0,0018	0,0233 ± 0,1203	0,004 ± 0,0219
(Bacillota) *Butyrivibrio crossotus*	0,0817 ± 0,3791	0,179 ± 0,6326	0,1016 ± 0,4327	0,0838 ± 0,2423
(Bacillota) *Butyrivibrio fibrisolvens*	0 ± 0	0,002 ± 0,0109	0 ± 0	0 ± 0
(Bacillota) *Caecibacter massiliensis*	0 ± 0	0,001 ± 0,0054	0 ± 0	0 ± 0
(Bacillota) *Caecibacterium sporoformans*	0 ± 0	0 ± 0	0 ± 0	0,0006 ± 0,0036
(Bacillota) *Caproiciproducens galactitolivorans*	0,0041 ± 0,0162	0,023 ± 0,0329	0,0097 ± 0,0209	0,0042 ± 0,0136
(Bacillota) *Carnobacterium divergens*	0 ± 0	0 ± 0	0 ± 0	0 ± 0
(Bacillota) *Carnobacterium maltaromaticum*	0 ± 0	0 ± 0	0 ± 0	0 ± 0
(Bacillota) *Casaltella massiliensis*	0 ± 0	0,0003 ± 0,0018	0 ± 0	0 ± 0
(Bacillota) *Catabacter hongkongensis*	0 ± 0	0 ± 0	0 ± 0	0,0018 ± 0,0102
(Bacillota) *Catenibacterium mitsuokai*	0,3133 ± 1,406	0,5683 ± 1,3292	0,4769 ± 1,4398	0,268 ± 1,0313
(Bacillota) *Cellulosilyticum lentocellum*	0 ± 0	0,0023 ± 0,0127	0 ± 0	0,0003 ± 0,0018

(Bacillota) *Cellulosilyticum ruminicola*	0 ± 0	0 ± 0	0 ± 0	0 ± 0
(Bacillota) *Christensenella hongkongensis*	0,0323 ± 0,1229	0,1 ± 0,2794	0,035 ± 0,1086	0,0119 ± 0,0383
(Bacillota) *Christensenella massiliensis*	0,012 ± 0,062	0,07 ± 0,1157	0,1205 ± 0,4427	0,0952 ± 0,4839
(Bacillota) *Christensenella minuta*	0,0315 ± 0,1197	0,0336 ± 0,0692	0,019 ± 0,0613	0,0339 ± 0,135
(Bacillota) *Christensenella timonensis*	0,0447 ± 0,2408	0,146 ± 0,449	0,0595 ± 0,2209	0,0349 ± 0,1754
(Bacillota) *Clostridioides difficile*	0 ± 0	0 ± 0	0,011 ± 0,0602	0 ± 0
(Bacillota) *Clostridium aldenense*	0 ± 0	0,001 ± 0,0054	0 ± 0	0 ± 0
(Bacillota) *Clostridium asparagiforme*	0 ± 0	0 ± 0	0 ± 0	0 ± 0
(Bacillota) *Clostridium baratii*	0,0336 ± 0,147	0,026 ± 0,1424	0 ± 0	0 ± 0
(Bacillota) *Clostridium bartlettii*	0,455 ± 2,4921	0 ± 0	0 ± 0	0 ± 0
(Bacillota) *Clostridium bolteae*	0 ± 0	0 ± 0	0 ± 0	0 ± 0
(Bacillota) *Clostridium butyricum*	0 ± 0	0,0016 ± 0,0091	0,0033 ± 0,0182	0,0469 ± 0,2534
(Bacillota) *Clostridium cadaveris*	0 ± 0	0 ± 0	0 ± 0	0 ± 0
(Bacillota) *Clostridium celatum*	0 ± 0	0 ± 0	0 ± 0	0,009 ± 0,0492
(Bacillota) *Clostridium citroniae*	0 ± 0	0 ± 0	0,0003 ± 0,0018	0 ± 0
(Bacillota) *Clostridium clostridioforme*	0 ± 0	0 ± 0	0,0023 ± 0,0127	0,006 ± 0,0328
(Bacillota) *Clostridium cocleatum*	0 ± 0	0 ± 0	0,008 ± 0,0438	0,021 ± 0,0804
(Bacillota) *Clostridium colinum*	0 ± 0	0 ± 0	0 ± 0	0 ± 0
(Bacillota) *Clostridium cylindrosporum*	0 ± 0	0 ± 0	0 ± 0	0 ± 0
(Bacillota) *Clostridium dakarense*	0 ± 0	0 ± 0	0 ± 0	0 ± 0
(Bacillota) *Clostridium Disporicum*	0,0683 ± 0,2127	0 ± 0	0 ± 0	0 ± 0
(Bacillota) *Clostridium glycyrrhizinilyticum*	0 ± 0	0 ± 0	0 ± 0	0 ± 0
(Bacillota) *Clostridium hiranonis*	0 ± 0	0 ± 0	0 ± 0	0 ± 0
(Bacillota) *Clostridium innocuum*	0,0813 ± 0,3282	0 ± 0	0,1536 ± 0,8266	0,019 ± 0,0546
(Bacillota) *Clostridium jeddahense*	0 ± 0	0,0036 ± 0,0154	0 ± 0	0 ± 0
(Bacillota) *Clostridium kluyveri*	0 ± 0	0 ± 0	0 ± 0	0 ± 0
(Bacillota) *Clostridium lactatifermentans*	0 ± 0	0 ± 0	0 ± 0	0 ± 0
(Bacillota) *Clostridium lavalense*	0 ± 0	0,005 ± 0,0273	0,0113 ± 0,0438	0,0086 ± 0,0474
(Bacillota) *Clostridium leptum*	0,0256 ± 0,1035	0 ± 0	0,0023 ± 0,0089	0,3232 ± 1,7311
(Bacillota) *Clostridium mayornbel*	0 ± 0	0 ± 0	0 ± 0	0 ± 0
(Bacillota) *Clostridium merdae*	0 ± 0	0,0113 ± 0,0223	0,0036 ± 0,0154	0,0207 ± 0,047
(Bacillota) *Clostridium methylpentosum*	0 ± 0	0 ± 0	0 ± 0	0,0435 ± 0,1683
(Bacillota) *Clostridium moniliforme*	0 ± 0	0 ± 0	0 ± 0	0 ± 0
(Bacillota) *Clostridium neonatale*	0 ± 0	0 ± 0	0 ± 0	0,0216 ± 0,1186
(Bacillota) *Clostridium paraputrificum*	0,0056 ± 0,031	0,0046 ± 0,0202	0,0033 ± 0,0149	0,0006 ± 0,0036
(Bacillota) *Clostridium perfringens*	0,5423 ± 2,076	0,6123 ± 2,0293	0,1076 ± 0,5784	0,0167 ± 0,0494
(Bacillota) *Clostridium phoceensis*	0 ± 0	0 ± 0	0 ± 0	0 ± 0
(Bacillota) *Clostridium quinii*	0 ± 0	0 ± 0	0 ± 0	0 ± 0

(Bacillota) *Clostridium ramosum*	0 ± 0	0 ± 0	0 ± 0	0 ± 0
(Bacillota) *Clostridium saccharobutylicum*	0 ± 0	0 ± 0	0 ± 0	0 ± 0
(Bacillota) *Clostridium saccharogumia*	0,0583 ± 0,3195	0 ± 0	0 ± 0	0 ± 0
(Bacillota) *Clostridium saccharolyticum*	0 ± 0	0 ± 0	0 ± 0	0 ± 0
(Bacillota) *Clostridium sartagoforme*	0 ± 0	0 ± 0	0 ± 0	0 ± 0
(Bacillota) *Clostridium saudiense*	0,2841 ± 0,7238	0,6713 ± 0,9365	0,6084 ± 0,9296	0,9532 ± 2,3244
(Bacillota) *Clostridium scindens*	0,01 ± 0,041	0 ± 0	0,0076 ± 0,0383	0,0298 ± 0,1123
(Bacillota) *Clostridium spiroforme*	0,02 ± 0,0555	0,0056 ± 0,031	0,004 ± 0,0184	0,004 ± 0,0219
(Bacillota) *Clostridium symbiosum*	0,015 ± 0,0595	0 ± 0	0 ± 0	0 ± 0
(Bacillota) *Clostridium tarantellae*	0 ± 0	0 ± 0	0 ± 0	0 ± 0
(Bacillota) *Clostridium uliginosum*	0 ± 0	0 ± 0	0 ± 0	0 ± 0
(Bacillota) *Clostridium ventriculi*	0 ± 0	0 ± 0	0 ± 0	0 ± 0
(Bacillota) *Clostridium vincentii*	0,0036 ± 0,02	0,001 ± 0,0054	0 ± 0	0,0117 ± 0,0644
(Bacillota) *Colidextribacter massiliensis*	0 ± 0	0 ± 0	0 ± 0	0 ± 0
(Bacillota) *Coprobacillus cateniformis*	0,0203 ± 0,1094	0,0046 ± 0,022	0,0912 ± 0,4055	0,0141 ± 0,0492
(Bacillota) *Coprococcus catus*	0,2069 ± 0,2495	0,153 ± 0,2063	0,2054 ± 0,3051	0,1488 ± 0,1877
(Bacillota) *Coprococcus comes*	0,6005 ± 0,6674	0,614 ± 0,9013	0,5699 ± 0,6631	0,5877 ± 0,6961
(Bacillota) *Coprococcus eutactus*	0,157 ± 0,545	0,6326 ± 1,0293	1,0926 ± 2,3421	0,9154 ± 2,6719
(Bacillota) *Coprococcus torques*	0 ± 0	0 ± 0	0 ± 0	0 ± 0
(Bacillota) *Criibacterium bergeronii*	0,017 ± 0,0931	0 ± 0	0 ± 0	0 ± 0
(Bacillota) *Cryptanaerobacter phenolicus*	0 ± 0	0 ± 0	0 ± 0	0 ± 0
(Bacillota) *Defluviitalea raffinosedens*	0 ± 0	0 ± 0	0 ± 0	0 ± 0
(Bacillota) *Defluviitalea saccharophila*	0 ± 0	0,008 ± 0,0184	0,003 ± 0,0095	0,0016 ± 0,0064
(Bacillota) *Desulfofarcimen acetoxidans*	0,001 ± 0,0056	0,0126 ± 0,0572	0 ± 0	0,0229 ± 0,0901
(Bacillota) *Desulfonispora thiosulfatigenes*	0 ± 0	0,0053 ± 0,0208	0 ± 0	0 ± 0
(Bacillota) *Desulfosporosinus auripigmenti*	0 ± 0	0,0003 ± 0,0018	0 ± 0	0 ± 0
(Bacillota) *Desulfosporosinus fructosivorans*	0 ± 0	0 ± 0	0 ± 0	0 ± 0
(Bacillota) *Desulfosporosinus lacus*	0 ± 0	0,003 ± 0,0095	0 ± 0	0 ± 0
(Bacillota) *Dethiobacter alkaliphilus*	0 ± 0	0 ± 0	0 ± 0	0 ± 0
(Bacillota) *Dethiosulfatibacter aminovorans*	0 ± 0	0 ± 0	0 ± 0	0,0005 ± 0,0029
(Bacillota) *Dialister invisus*	0,1779 ± 0,4711	0,3816 ± 1,0137	0,2384 ± 0,4408	0,3386 ± 0,6627
(Bacillota) *Dialister micraerophilus*	0 ± 0	0 ± 0	0 ± 0	0 ± 0
(Bacillota) *Dialister pneumosintes*	0 ± 0	0 ± 0	0 ± 0	0 ± 0
(Bacillota) *Dialister propionicifaciens*	0,0011 ± 0,006	0 ± 0	0 ± 0	0 ± 0
(Bacillota) *Dialister succinatiphilus*	0,1698 ± 0,4118	0,4713 ± 0,815	0,3024 ± 0,8415	0,1374 ± 0,4315
(Bacillota) *Dielma fastidiosa*	0,0023 ± 0,0129	0 ± 0	0 ± 0	0,018 ± 0,0828
(Bacillota) *Dorea Dorea*	0 ± 0	0 ± 0	0 ± 0	0 ± 0
(Bacillota) *Dorea formicigenerans*	0,7113 ± 0,7779	0,137 ± 0,4471	0,1398 ± 0,2505	0,2365 ± 0,4814

(Bacillota) *Dorea longicatena*	2,3257 ± 1,8051	2,329 ± 1,6578	2,0413 ± 1,8984	1,8594 ± 1,5267
(Bacillota) *Dorea massiliensis*	0 ± 0	0 ± 0	0 ± 0	0,0235 ± 0,0938
(Bacillota) *Drancourtella massiliensis*	0,0307 ± 0,1357	0,2133 ± 0,7299	0,0057 ± 0,0159	0,011 ± 0,035
(Bacillota) *Eisenbergiella massiliensis*	0,0433 ± 0,2373	0 ± 0	0,0303 ± 0,1334	0,051 ± 0,2793
(Bacillota) *Eisenbergiella tayi*	0,0956 ± 0,5034	0 ± 0	0,0023 ± 0,0127	0,001 ± 0,0054
(Bacillota) *Emergencia timonensis*	0,0012 ± 0,0065	0,0356 ± 0,0594	0,0143 ± 0,037	0,0102 ± 0,0277
(Bacillota) *Enterocloster aldenensis*	0 ± 0	0,002 ± 0,008	0 ± 0	0 ± 0
(Bacillota) *Enterocloster asparagiformis*	0 ± 0	0,0016 ± 0,0091	0 ± 0	0 ± 0
(Bacillota) *Enterocloster bolteae*	0,0561 ± 0,1959	0,4436 ± 1,0341	0,1234 ± 0,2953	0,0607 ± 0,1483
(Bacillota) *Enterocloster citroniae*	0 ± 0	0,009 ± 0,0265	0,0096 ± 0,0408	0,0985 ± 0,3309
(Bacillota) *Enterocloster clostridioformis*	0,0003 ± 0,0018	0,059 ± 0,1364	0,0113 ± 0,0354	0,0153 ± 0,0588
(Bacillota) *Enterococcus avium*	0,0013 ± 0,0073	0 ± 0	0 ± 0	0 ± 0
(Bacillota) *Enterococcus casseliflavus*	0 ± 0	0 ± 0	0 ± 0	0,019 ± 0,104
(Bacillota) *Enterococcus cecorum*	0,0113 ± 0,062	0 ± 0	0 ± 0	0 ± 0
(Bacillota) *Enterococcus durans*	33,5566 ± 183,6652	0,0123 ± 0,0305	0,0162 ± 0,0805	0,0113 ± 0,0548
(Bacillota) *Enterococcus faecalis*	0,2601 ± 1,1986	0,0583 ± 0,1732	0,0036 ± 0,02	0 ± 0
(Bacillota) *Enterococcus faecium*	0,188 ± 0,6249	0,5216 ± 1,134	0,0096 ± 0,0529	0,1126 ± 0,4366
(Bacillota) *Enterococcus gallinarum*	0 ± 0	0 ± 0	0,0006 ± 0,0036	0 ± 0
(Bacillota) *Enterococcus hermanniensis*	0 ± 0	0,0033 ± 0,0182	0 ± 0	0 ± 0
(Bacillota) *Enterococcus hirae*	0,011 ± 0,0602	0,0566 ± 0,2132	0,0005 ± 0,0027	0,001 ± 0,0054
(Bacillota) *Enterococcus lemanii*	0 ± 0	0 ± 0	0 ± 0	0 ± 0
(Bacillota) *Enterococcus mundtii*	0 ± 0	0 ± 0	0 ± 0	0 ± 0
(Bacillota) *Enterococcus phoeniculicola*	0 ± 0	0 ± 0	0 ± 0	0 ± 0
(Bacillota) *Enterococcus pseudoavium*	0 ± 0	0 ± 0	0 ± 0	0 ± 0
(Bacillota) *Enterococcus raffnosus*	0 ± 0	0 ± 0	0 ± 0	0 ± 0
(Bacillota) *Enterococcus saccharolyticus*	0 ± 0	0 ± 0	0 ± 0	0 ± 0
(Bacillota) *Enterococcus termitis*	0 ± 0	0 ± 0	0 ± 0	0 ± 0
(Bacillota) *Enterococcus thailandicus*	0 ± 0	0,001 ± 0,0054	0 ± 0	0 ± 0
(Bacillota) *Enterococcus villorum*	0 ± 0	0,001 ± 0,0054	0 ± 0	0 ± 0
(Bacillota) *Ercella succinigenes*	0 ± 0	0 ± 0	0 ± 0	0 ± 0
(Bacillota) *Erysipelatoclostridium ramosum*	0,1017 ± 0,3257	0,0793 ± 0,1924	0,1491 ± 0,4886	0,0903 ± 0,3062
(Bacillota) *Erysipelothrix inopinata*	0 ± 0	0 ± 0	0,0006 ± 0,0036	0 ± 0
(Bacillota) *Erysipelothrix larvae*	0 ± 0	0 ± 0	0 ± 0	0 ± 0
(Bacillota) *Erysipelothrix tonsillarum*	0 ± 0	0 ± 0	0 ± 0	0 ± 0
(Bacillota) *Ethanoligenens harbinense*	0 ± 0	0,0963 ± 0,5257	0 ± 0	0,028 ± 0,1533
(Bacillota) *Eubacterium biforme*	0 ± 0	0 ± 0	0 ± 0	0 ± 0
(Bacillota) *Eubacterium callander*	0 ± 0	0 ± 0	0 ± 0	0 ± 0
(Bacillota) *Eubacterium callanderi*	0 ± 0	0,0033 ± 0,0109	0 ± 0	0 ± 0

(Bacillota) *Eubacterium coprostanoligenes*	0,1787 ± 0,4154	1,2096 ± 0,8979	0,8747 ± 1,4638	0,5669 ± 1,3361
(Bacillota) *Eubacterium cylindroides*	0 ± 0	0 ± 0	0 ± 0	0 ± 0
(Bacillota) *Eubacterium dolichum*	0 ± 0	0 ± 0	0 ± 0	0,1254 ± 0,6868
(Bacillota) *Lachnospira eligens*	0,3223 ± 0,8822	0,0696 ± 0,3815	0,0816 ± 0,3458	0,0823 ± 0,3475
(Bacillota) *Anaerostipes hadrus*	0,2093 ± 1,1465	0,1366 ± 0,7485	0 ± 0	0,7879 ± 2,4919
(Bacillota) *Eubacterium hadrus*	#DIV/0!	0 ± 0	0 ± 0	0 ± 0
(Bacillota) *Anaerobutyricum hallii*	1,9426 ± 2,3866	0 ± 0	0,3206 ± 0,6614	0,393 ± 0,8852
(Bacillota) *Eubacterium limosum*	0 ± 0	0 ± 0	0 ± 0	0 ± 0
(Bacillota) *Eubacterium oxidoreducens*	0,0051 ± 0,0162	0,2353 ± 0,5857	0,0558 ± 0,12	0,1745 ± 0,5417
(Bacillota) *Eubacterium ramulus*	0,118 ± 0,179	0,2153 ± 0,3301	0,108 ± 0,1747	0,0697 ± 0,1507
(Bacillota) *Eubacterium recale*	0,1146 ± 0,4465	0 ± 0	0 ± 0	0,1017 ± 0,5203
(Bacillota) *Agathobacter rectalis*	1,7243 ± 2,7026	0 ± 0	0,545 ± 1,8623	0,559 ± 2,5295
(Bacillota) *Eubacterium ruminantium*	0,0301 ± 0,1215	0,171 ± 0,3909	0,2464 ± 0,6152	0,199 ± 0,7658
(Bacillota) *Eubacterium siraeum*	0 ± 0	0 ± 0	0 ± 0	0 ± 0
(Bacillota) *Eubacterium uniforme*	0 ± 0	0,0016 ± 0,0091	0,0013 ± 0,0073	0 ± 0
(Bacillota) *Eubacterium xylanophilum*	0,0226 ± 0,1241	0,076 ± 0,1441	0,0554 ± 0,1315	0,0416 ± 0,1102
(Bacillota) *Extibacter muris*	0 ± 0	0 ± 0	0 ± 0	0 ± 0
(Bacillota) *Facklamia hominis*	0 ± 0	0 ± 0	0 ± 0	0 ± 0
(Bacillota) *Faecalibacterium prausnitzii*	6,957 ± 7,9214	8,2613 ± 5,5485	10,7851 ± 5,3911	9,0434 ± 6,2528
(Bacillota) *Faecalicatena contorta*	0,0861 ± 0,2281	0,5413 ± 0,5023	0,2734 ± 0,4521	0,2433 ± 0,384
(Bacillota) *Faecalicatena fissicatena*	0,0494 ± 0,161	0,2133 ± 0,598	0,0416 ± 0,1532	0,0277 ± 0,144
(Bacillota) *Faecalicatena orotica*	0 ± 0	0,0106 ± 0,0185	0,0113 ± 0,053	0,0351 ± 0,1522
(Bacillota) *Faecalicoccus acidiformans*	0 ± 0	0,0103 ± 0,0241	0,0013 ± 0,005	0,0018 ± 0,0102
(Bacillota) *Faecalicoccus plemorphus*	0 ± 0	0 ± 0	0 ± 0	0 ± 0
(Bacillota) *Faecalicoccus pleomorphus*	0 ± 0	0 ± 0	0 ± 0	0 ± 0
(Bacillota) *Faecalimonas umbilicata*	0,0286 ± 0,157	0,283 ± 0,5415	0,0612 ± 0,1305	0,1499 ± 0,3401
(Bacillota) *Faecalitalea cylindroides*	0,0253 ± 0,1389	0,0463 ± 0,2154	0 ± 0	0,0206 ± 0,1131
(Bacillota) *Fastidiosipila sanguinis*	0 ± 0	0 ± 0	0 ± 0	0 ± 0
(Bacillota) *Fenollaria timonensis*	0 ± 0	0 ± 0	0 ± 0	0 ± 0
(Bacillota) *Finegoldia Magna*	0 ± 0	0,0006 ± 0,0036	0 ± 0	0 ± 0
(Bacillota) *Flavonifractor plautii*	0,2365 ± 0,4021	0,7066 ± 1,6466	0,2956 ± 0,3356	0,291 ± 0,5044
(Bacillota) *Flintibacter butyricus*	0,1163 ± 0,6371	0,2083 ± 1,141	0 ± 0	0,0004 ± 0,0025
(Bacillota) *Floricoccus penangensis*	0 ± 0	0 ± 0	0 ± 0	0 ± 0
(Bacillota) *Frisingicoccus caecimuris*	0,0091 ± 0,0502	0,019 ± 0,0474	0,0036 ± 0,0144	0,0175 ± 0,0652
(Bacillota) *Fusibacter fontis*	0 ± 0	0,0003 ± 0,0018	0 ± 0	0 ± 0
(Bacillota) *Fusicatenibacter saccharivorans*	1,0887 ± 1,6955	1,2546 ± 1,9095	2,2616 ± 2,0472	1,6989 ± 1,8016
(Bacillota) *Gemella sanguinis*	0 ± 0	0 ± 0	0 ± 0	0 ± 0
(Bacillota) *Globicatella sulfidifaciens*	0 ± 0	0,0076 ± 0,0419	0 ± 0	0 ± 0

(Bacillota) *Gracilibacter thermotolerans*	0 ± 0	0 ± 0	0 ± 0	0 ± 0
(Bacillota) *Granulicatella adiacens*	0,005 ± 0,0273	0,0106 ± 0,0302	0 ± 0	0,004 ± 0,0219
(Bacillota) *Granulicatella elegans*	0 ± 0	0 ± 0	0,0003 ± 0,0018	0 ± 0
(Bacillota) *Guggenheimella bovis*	0 ± 0	0,0076 ± 0,0367	0 ± 0	0 ± 0
(Bacillota) *Haloimpatiens lingqiaonensis*	0 ± 0	0 ± 0	0,0433 ± 0,2373	0 ± 0
(Bacillota) *Harryflintia acetispora*	0,024 ± 0,1277	0,109 ± 0,2112	0,024 ± 0,1062	0,0108 ± 0,0292
(Bacillota) *Holdemanella biformis*	0,357 ± 1,1373	1,1343 ± 2,4272	0,4774 ± 1,0307	0,4726 ± 1,1074
(Bacillota) *Holdemania filiformis*	0 ± 0	0,015 ± 0,0293	0,0059 ± 0,0126	0,0043 ± 0,0138
(Bacillota) *Holdemania massiliensis*	0,002 ± 0,0113	0,0033 ± 0,0109	0 ± 0	0,0013 ± 0,0073
(Bacillota) *Howardella ureilytica*	0 ± 0	0 ± 0	0 ± 0	0 ± 0
(Bacillota) *Hungatella effluvi*	0 ± 0	0,0233 ± 0,1278	0 ± 0	0 ± 0
(Bacillota) *Hungatella effluvii*	0 ± 0	0,001 ± 0,0054	0 ± 0	0 ± 0
(Bacillota) *Hungatella hathewayi*	0,0696 ± 0,2799	0,0026 ± 0,0146	0,0423 ± 0,2101	0 ± 0
(Bacillota) *Hungatella xylanolytica*	0,0075 ± 0,041	0,007 ± 0,0383	0 ± 0	0 ± 0
(Bacillota) *Hydrogenoanaerobacterium saccharovorans*	0 ± 0	0,002 ± 0,0092	0,002 ± 0,0081	0 ± 0
(Bacillota) *Ileibacterium valens*	0 ± 0	0 ± 0	0 ± 0	0 ± 0
(Bacillota) *Intestinibacillus massiliensis*	0,0016 ± 0,0091	0,013 ± 0,0515	0,0225 ± 0,0861	0,0006 ± 0,0036
(Bacillota) *Intestinibacter bartlettii*	0,2727 ± 0,4788	0,7296 ± 0,8738	0,2477 ± 0,4736	0,2833 ± 0,6417
(Bacillota) *Intestinimonas butyriciproducens*	0,0076 ± 0,0367	0 ± 0	0,0043 ± 0,0237	0,0063 ± 0,0346
(Bacillota) *Intestinimonas gabonensis*	0 ± 0	0 ± 0	0 ± 0	0,0771 ± 0,4226
(Bacillota) *Intestinimonas timonensis*	0 ± 0	0 ± 0	0 ± 0	0 ± 0
(Bacillota) *Kineothrix alysoides*	0,0488 ± 0,2344	0,3083 ± 0,5122	0,1067 ± 0,3275	0,0724 ± 0,2484
(Bacillota) *Lachnoanaerobaculum orale*	0 ± 0	0,0003 ± 0,0018	0 ± 0	0 ± 0
(Bacillota) *Lachnobacterium bovis*	0 ± 0	0 ± 0	0 ± 0	0 ± 0
(Bacillota) *Lachnoclostridium clostridioforme*	0,0606 ± 0,263	0 ± 0	0,0126 ± 0,0693	0,002 ± 0,008
(Bacillota) *Lachnoclostridium pacaense*	0,0604 ± 0,2815	0,2306 ± 0,4917	0,0527 ± 0,1776	0,2305 ± 0,7813
(Bacillota) *Lachnospira eligens*	0,0717 ± 0,2344	0,5146 ± 0,8247	0,263 ± 0,9589	0,2692 ± 0,7542
(Bacillota) *Lachnospira multipara*	0 ± 0	0,0023 ± 0,0127	0 ± 0	0 ± 0
(Bacillota) *Lachnospira pectinoschiza*	0,1286 ± 0,3366	0,1853 ± 0,4057	0,3156 ± 0,5539	0,0855 ± 0,2758
(Bacillota) *Lachnotalea glycerini*	0 ± 0	0,005 ± 0,0238	0 ± 0	0 ± 0
(Bacillota) *Lacrimispora aerotolerans*	0 ± 0	0 ± 0	0,003 ± 0,0164	0 ± 0
(Bacillota) *Lacrimispora algidixylanolytica*	0 ± 0	0 ± 0	0 ± 0	0 ± 0
(Bacillota) *Lacrimispora amygdalina*	0,0193 ± 0,082	0,112 ± 0,2405	0,1648 ± 0,3876	0,0769 ± 0,2034
(Bacillota) *Lacrimispora celerecrescens*	0 ± 0	0,1376 ± 0,5897	0,0003 ± 0,0018	0,163 ± 0,6811
(Bacillota) *Lacrimispora indolis*	0,004 ± 0,0201	0,0236 ± 0,0632	0,001 ± 0,004	0,6309 ± 2,294
(Bacillota) *Lacrimispora saccharolytica*	0,0079 ± 0,0434	0,098 ± 0,4017	0,0018 ± 0,0077	0,0571 ± 0,22
(Bacillota) *Lacrimispora sphenoides*	0,0069 ± 0,0381	0,0833 ± 0,1425	0,2327 ± 0,5715	0,1127 ± 0,2419
(Bacillota) *Lacrimispora xylanolytica*	0 ± 0	0,0423 ± 0,1126	0 ± 0	0,002 ± 0,0066

(Bacillota) *Lacrimospora saccharolytica*	0 ± 0	0 ± 0	0 ± 0	0 ± 0
(Bacillota) *Lacticaseibacillus pantheris*	0 ± 0	0 ± 0	0 ± 0	0 ± 0
(Bacillota) *Lacticaseibacillus paracasei*	0,0097 ± 0,0531	0,0006 ± 0,0036	0,0003 ± 0,0018	0,0006 ± 0,0036
(Bacillota) *Lacticaseibacillus rhamnosus*	0 ± 0	0,0346 ± 0,1642	0,005 ± 0,0273	0 ± 0
(Bacillota) *Lacticaseibacillus zeae*	0 ± 0	0 ± 0	0 ± 0	0 ± 0
(Bacillota) *Lactiplantibacillus pentosus*	0 ± 0	0 ± 0	0 ± 0	0 ± 0
(Bacillota) *Lactiplantibacillus plantarum*	0,0166 ± 0,0912	0 ± 0	0 ± 0	0 ± 0
(Bacillota) *Lactobacillus acidophilus*	0,003 ± 0,0164	0,0023 ± 0,0127	0,003 ± 0,0164	0,024 ± 0,1314
(Bacillota) *Lactobacillus amylovorus*	0,0051 ± 0,0281	0,0066 ± 0,0242	0,021 ± 0,115	0,0146 ± 0,0803
(Bacillota) *Lactobacillus antri*	0 ± 0	0 ± 0	0 ± 0	0 ± 0
(Bacillota) *Lactobacillus casei*	0 ± 0	0 ± 0	0,0023 ± 0,0127	0 ± 0
(Bacillota) *Lactobacillus colini*	0,0006 ± 0,0036	0,0043 ± 0,0143	0 ± 0	0 ± 0
(Bacillota) *Lactobacillus crispatus*	0,4496 ± 1,965	0,1603 ± 0,559	0,0071 ± 0,039	0 ± 0
(Bacillota) *Lactobacillus curvatus*	0 ± 0	0 ± 0	0 ± 0	0 ± 0
(Bacillota) *Lactobacillus delbrueckii*	0,0444 ± 0,2225	0,0053 ± 0,0163	0,003 ± 0,0105	0,0026 ± 0,0146
(Bacillota) *Lactobacillus fornicalis*	0 ± 0	0,004 ± 0,0219	0 ± 0	0 ± 0
(Bacillota) *Lactobacillus gallinarum*	0 ± 0	0,0003 ± 0,0018	0 ± 0	0 ± 0
(Bacillota) *Lactobacillus gasseri*	0,3113 ± 0,8283	0,7916 ± 2,0252	0,0016 ± 0,0091	0,0006 ± 0,0036
(Bacillota) *Lactobacillus helsingborgensis*	0 ± 0	0 ± 0	0 ± 0	0,0003 ± 0,0018
(Bacillota) *Lactobacillus helveticus*	0 ± 0	0 ± 0	0 ± 0	0 ± 0
(Bacillota) *Lactobacillus iners*	0 ± 0	0 ± 0	0 ± 0	0 ± 0
(Bacillota) *Lactobacillus jensenii*	0 ± 0	0,0003 ± 0,0018	0 ± 0	0 ± 0
(Bacillota) *Lactobacillus johnsonii*	0,0016 ± 0,0091	0,0373 ± 0,1651	0 ± 0	0 ± 0
(Bacillota) *Lactobacillus oris*	0,035 ± 0,1581	0 ± 0	0 ± 0	0 ± 0
(Bacillota) *Lactobacillus paragasseri*	0 ± 0	0 ± 0	0 ± 0	0,0006 ± 0,0036
(Bacillota) *Lactobacillus paraplantarum*	0 ± 0	0 ± 0	0 ± 0	0 ± 0
(Bacillota) *Lactiplantibacillus plantarum*	0 ± 0	0 ± 0	0 ± 0	0 ± 0
(Bacillota) *Lacticaseibacillus rhamnosus*	0,0013 ± 0,0073	0 ± 0	0 ± 0	0,003 ± 0,0164
(Bacillota) *Lactobacillus rodentium*	0 ± 0	0 ± 0	0 ± 0	0 ± 0
(Bacillota) *Lactobacillus rogosae*	0,0076 ± 0,0419	0,119 ± 0,1788	0,6121 ± 1,2118	0,1971 ± 0,4526
(Bacillota) *Lactobacillus taiwanensis*	0,0006 ± 0,0036	0,007 ± 0,0211	0 ± 0	0 ± 0
(Bacillota) *Lactobacillus vaginalis*	0,0166 ± 0,076	0 ± 0	0 ± 0	0 ± 0
(Bacillota) *Lactococcus cremoris*	0 ± 0	0,0003 ± 0,0018	0 ± 0	0 ± 0
(Bacillota) *Lactococcus garvieae*	0,0036 ± 0,02	0,0283 ± 0,1327	0 ± 0	0 ± 0
(Bacillota) *Lactococcus lactis*	0,0358 ± 0,1708	0,06 ± 0,1775	0,0086 ± 0,0354	0,007 ± 0,0383
(Bacillota) *Lactococcus laudensis*	0 ± 0	0 ± 0	0 ± 0	0 ± 0
(Bacillota) *Lactococcus piscium*	0,001 ± 0,0054	0 ± 0	0 ± 0	0,0003 ± 0,0018
(Bacillota) *Lactococcus plantarum*	0 ± 0	0,0003 ± 0,0018	0 ± 0	0 ± 0

(Bacillota) *Lactococcus raffinolactis*	0 ± 0	0,0003 ± 0,0018	0 ± 0	0 ± 0
(Bacillota) *Lactococcus taiwanensis*	0 ± 0	0 ± 0	0 ± 0	0 ± 0
(Bacillota) *Lactonifactor longoviformis*	0 ± 0	0,0003 ± 0,0018	0 ± 0	0 ± 0
(Bacillota) *Latilactobacillus curvatus*	0 ± 0	0 ± 0	0,0003 ± 0,0018	0,001 ± 0,0054
(Bacillota) *Latilactobacillus graminis*	0 ± 0	0 ± 0	0 ± 0	0 ± 0
(Bacillota) *Latilactobacillus sakei*	0,0635 ± 0,2825	0,059 ± 0,2807	0 ± 0	0,0106 ± 0,0494
(Bacillota) *Lawsonibacter asaccharolyticus*	0 ± 0	0 ± 0	0,0096 ± 0,0407	0,015 ± 0,0515
(Bacillota*) Lcatobacillus mucosae*	0 ± 0	0 ± 0	0 ± 0	0 ± 0
(Bacillota) *Lentilactobacillus kefiri*	0 ± 0	0 ± 0	0 ± 0	0 ± 0
(Bacillota) *Leuconostoc carnosum*	0 ± 0	0 ± 0	0 ± 0	0 ± 0
(Bacillota) *Leuconostoc citreum*	0 ± 0	0,006 ± 0,0256	0 ± 0	0 ± 0
(Bacillota) *Leuconostoc garlicum*	0,0016 ± 0,0091	0 ± 0	0 ± 0	0 ± 0
(Bacillota) *Leuconostoc gelidum*	0 ± 0	0,015 ± 0,0682	0 ± 0	0,0003 ± 0,0018
(Bacillota) *Leuconostoc kimchii*	0 ± 0	0 ± 0	0 ± 0	0 ± 0
(Bacillota) *Leuconostoc lactis*	0 ± 0	0 ± 0	0 ± 0	0 ± 0
(Bacillota) *Leuconostoc mesenteroides*	0,002 ± 0,0109	0,0033 ± 0,0118	0 ± 0	0,0003 ± 0,0018
(Bacillota) *Leuconostoc pseudomesenteroides*	0 ± 0	0 ± 0	0 ± 0	0 ± 0
(Bacillota) *Levilactobacillus brevis*	0 ± 0	0 ± 0	0 ± 0	0 ± 0
(Bacillota) *Ligilactobacillus apodemi*	0 ± 0	0 ± 0	0 ± 0	0 ± 0
(Bacillota) *Ligilactobacillus ruminis*	0,0306 ± 0,1679	0,045 ± 0,2464	0,0206 ± 0,1024	0,293 ± 1,5935
(Bacillota) *Ligilactobacillus salivarius*	0 ± 0	0,0966 ± 0,4724	0,002 ± 0,0109	0 ± 0
(Bacillota) *Limosilactobacillus caviae*	0 ± 0	0 ± 0	0 ± 0	0 ± 0
(Bacillota) *Limosilactobacillus coleohominis*	0 ± 0	0 ± 0	0 ± 0	0 ± 0
(Bacillota) *Limosilactobacillus fermentum*	0,069 ± 0,2098	0,2123 ± 0,6143	0,003 ± 0,0164	0 ± 0
(Bacillota) *Limosilactobacillus frumenti*	0,0003 ± 0,0018	0,001 ± 0,004	0 ± 0	0 ± 0
(Bacillota) *Limosilactobacillus gorillae*	0 ± 0	0 ± 0	0 ± 0	0 ± 0
(Bacillota) *Limosilactobacillus mucosae*	0,3907 ± 2,1133	0,006 ± 0,0328	0 ± 0	0 ± 0
(Bacillota) *Limosilactobacillus oris*	0,0259 ± 0,1418	0,0363 ± 0,1878	0,0028 ± 0,0111	0,001 ± 0,0054
(Bacillota) *Limosilactobacillus panis*	0 ± 0	0 ± 0	0 ± 0	0 ± 0
(Bacillota) *Limosilactobacillus reuteri*	0,0083 ± 0,0456	0,0753 ± 0,3551	0 ± 0	0 ± 0
(Bacillota) *Limosilactobacillus vaginalis*	0 ± 0	0,1953 ± 0,9046	0 ± 0	0 ± 0
(Bacillota) *Loigolactobacillus bifermentans*	0 ± 0	0 ± 0	0 ± 0	0 ± 0
(Bacillota) *Longibaculum muris*	0,038 ± 0,2081	0,644 ± 1,3071	0,557 ± 0,9177	0,6703 ± 1,0057
(Bacillota) *Longicatena caecimuris*	0,0014 ± 0,008	0,06 ± 0,1284	0,0095 ± 0,0359	0,022 ± 0,0595
(Bacillota) *Lutispora thermophila*	0 ± 0	0 ± 0	0,004 ± 0,0184	0,0805 ± 0,441
(Bacillota) *Mageeibacillus indolicus*	0 ± 0	0 ± 0	0,001 ± 0,0054	0,1962 ± 0,76
(Bacillota) *Mammaliicoccus vitulinus*	0 ± 0	0 ± 0	0 ± 0	0 ± 0
(Bacillota) *Marseillibacter massiliensis*	0 ± 0	0 ± 0	0 ± 0	0 ± 0

(Bacillota) *Marvinbryantia formatexigens*	0 ± 0	0 ± 0	0 ± 0	0 ± 0
(Bacillota) *Massilimicrobiota timonensis*	0 ± 0	0,0083 ± 0,0265	0,0076 ± 0,026	0,016 ± 0,0495
(Bacillota) *Massilioclostridium coli*	0 ± 0	0 ± 0	0 ± 0	0 ± 0
(Bacillota) *Massiliomicrobiota timonensis*	0 ± 0	0,001 ± 0,0054	0 ± 0	0 ± 0
(Bacillota) *Mediterraneibacter faecis*	0,147 ± 0,4627	0,8993 ± 1,041	0,3634 ± 0,5652	0,3764 ± 0,9563
(Bacillota) *Mediterraneibacter glycyrrhizinilyticus*	0,0013 ± 0,0073	0,0513 ± 0,1467	0,0116 ± 0,032	0,1574 ± 0,6846
(Bacillota) *Megamonas funiformis*	0 ± 0	0,0003 ± 0,0018	0,0253 ± 0,1332	0,0006 ± 0,0036
(Bacillota) *Megamonas hypermegale*	0 ± 0	0 ± 0	0,001 ± 0,0054	0,0043 ± 0,0237
(Bacillota) *Megamonas rupellensis*	0 ± 0	0,115 ± 0,6	0 ± 0	0,104 ± 0,5696
(Bacillota) *Megasphaera elsdenii*	0,019 ± 0,1003	0,0086 ± 0,0365	0,118 ± 0,501	0,009 ± 0,0492
(Bacillota) *Megasphaera hexanoica*	0 ± 0	0 ± 0	0,0013 ± 0,0073	0 ± 0
(Bacillota) *Megasphaera hominis*	0 ± 0	0 ± 0	0 ± 0	0 ± 0
(Bacillota) *Megasphaera indica*	0 ± 0	0 ± 0	0,001 ± 0,0054	0,0223 ± 0,1223
(Bacillota) *Megasphaera massiliensis*	0 ± 0	0,0073 ± 0,0401	0 ± 0	0 ± 0
(Bacillota) *Megasphaera micronuciformis*	0,004 ± 0,0219	0,001 ± 0,0054	0 ± 0	0 ± 0
(Bacillota) *Merdibacter massiliensis*	0 ± 0	0 ± 0	0 ± 0	0 ± 0
(Bacillota) *Merdimonas faecis*	0,0107 ± 0,0587	0,019 ± 0,0702	0 ± 0	0,0013 ± 0,0073
(Bacillota) *Mitsuokella jalaludinii*	0 ± 0	0,021 ± 0,0796	0,0636 ± 0,294	0 ± 0
(Bacillota) *Mitsuokella multacida*	0 ± 0	0,0033 ± 0,0182	0,0186 ± 0,1022	0 ± 0
(Bacillota) *Mobilitalea Sibirica*	0 ± 0	0,007 ± 0,0233	0,0023 ± 0,0127	0,001 ± 0,004
(Bacillota) *Mogibacterium diversum*	0,0023 ± 0,0127	0 ± 0	0 ± 0	0 ± 0
(Bacillota) *Mogibacterium neglectum*	0 ± 0	0,0343 ± 0,126	0,0003 ± 0,0018	0,001 ± 0,0054
(Bacillota) *Mogibacterium pumilum*	0 ± 0	0 ± 0	0 ± 0	0 ± 0
(Bacillota) *Mogibacterium timidum*	0 ± 0	0 ± 0	0 ± 0	0 ± 0
(Bacillota) *Monoglobus pectinilyticus*	0,06 ± 0,1581	0,4556 ± 1,2353	0,3487 ± 0,522	0,3399 ± 0,5829
(Bacillota) *Mordavella massiliensis*	0,0038 ± 0,0183	0,013 ± 0,0567	0,001 ± 0,004	0,0036 ± 0,0127
(Bacillota) *Moryella indoligenes*	0 ± 0	0 ± 0	0 ± 0	0,0809 ± 0,3141
(Bacillota) *Murdochiella massiliensis*	0 ± 0	0 ± 0	0 ± 0	0 ± 0
(Bacillota) *Murimonas intestini*	0,0006 ± 0,0036	0 ± 0	0 ± 0	0 ± 0
(Bacillota) *Negativibacillus massiliensis*	0 ± 0	0 ± 0	0 ± 0	0 ± 0
(Bacillota) *Negativicoccus massiliensis*	0 ± 0	0 ± 0	0 ± 0	0 ± 0
(Bacillota) *Nosocomiicoccus ampullae*	0 ± 0	0 ± 0	0 ± 0	0 ± 0
(Bacillota) *Nosocomiicoccus massiliensis*	0 ± 0	0 ± 0	0 ± 0	0 ± 0
(Bacillota) *Orenia metallireducens*	0 ± 0	0 ± 0	0 ± 0	0 ± 0
(Bacillota) *Oribacterium asaccharolyticum*	0 ± 0	0,0006 ± 0,0036	0 ± 0	0 ± 0
(Bacillota) *Oribacterium sinus*	0 ± 0	0,004 ± 0,0219	0 ± 0	0 ± 0
(Bacillota) *Oscilibacter massiliensis*	0 ± 0	0 ± 0	0 ± 0	0 ± 0
(Bacillota) *Oscillibacter ruminantium*	0,097 ± 0,2994	0,5796 ± 0,8144	0,4811 ± 0,8341	0,5747 ± 1,1664

(Bacillota) *Oscillibacter valericigenes*	0,5017 ± 1,4513	1,677 ± 1,491	1,0564 ± 1,6044	1,1881 ± 2,0613
(Bacillota) *Oxobacter pfennigii*	0 ± 0	0 ± 0	0,0006 ± 0,0036	0 ± 0
(Bacillota) *Paeniclostridium sordellii*	0,106 ± 0,5805	0,106 ± 0,5805	0 ± 0	0 ± 0
(Bacillota) *Paludicola psychrotolerans*	0 ± 0	0,024 ± 0,1092	0,0003 ± 0,0018	0 ± 0
(Bacillota) *Papillibacter cinnamivorans*	0,0152 ± 0,0637	0,0333 ± 0,0873	0,0068 ± 0,0259	0,006 ± 0,0265
(Bacillota) *Paraclostridium benzoelyticum*	0 ± 0	0 ± 0	0 ± 0	0 ± 0
(Bacillota) *Paraclostridium bifermentans*	0 ± 0	0 ± 0	0 ± 0	0 ± 0
(Bacillota) *Parasporobacterium paucivorans*	0 ± 0	0,0043 ± 0,0185	0 ± 0	0,0003 ± 0,0018
(Bacillota) *Parvimonas micra*	0 ± 0	0,0026 ± 0,0073	0,0006 ± 0,0036	0,0006 ± 0,0036
(Bacillota) *Paucilactobacillus oligofermentans*	0 ± 0	0 ± 0	0 ± 0	0 ± 0
(Bacillota) *Pediococcus acidilactici*	0,0003 ± 0,0018	0,0003 ± 0,0018	0 ± 0	0 ± 0
(Bacillota) *Pediococcus damnosu*	0 ± 0	0 ± 0	0 ± 0	0 ± 0
(Bacillota) *Pediococcus pentosaceus*	0 ± 0	0,001 ± 0,0054	0 ± 0	0 ± 0
(Bacillota) *Pelospora glutarica*	0 ± 0	0 ± 0	0 ± 0	0 ± 0
(Bacillota) *Pelotomaculum propionicicum*	0 ± 0	0 ± 0	0 ± 0	0 ± 0
(Bacillota) *Peptacetobacter hiranonis*	0,0097 ± 0,0534	0,002 ± 0,0092	0,0016 ± 0,0064	0,001 ± 0,0054
(Bacillota) *Peptococcus niger*	0,0221 ± 0,1212	0,0453 ± 0,1361	0,0093 ± 0,0457	0,0113 ± 0,062
(Bacillota) *Peptoniphilus asaccharolyticus*	0 ± 0	0 ± 0	0,0021 ± 0,0118	0,1352 ± 0,5935
(Bacillota) *Peptoniphilus coxii*	0 ± 0	0 ± 0	0,0005 ± 0,0031	0 ± 0
(Bacillota) *Peptoniphilus gorbachi*	0 ± 0	0 ± 0	0 ± 0	0 ± 0
(Bacillota) *Peptoniphilus grossensis*	0 ± 0	0,005 ± 0,0273	0 ± 0	0,0019 ± 0,0075
(Bacillota) *Peptoniphilus harei*	0,0063 ± 0,0346	0 ± 0	0 ± 0	0 ± 0
(Bacillota) *Peptoniphilus indolicus*	0 ± 0	0 ± 0	0 ± 0	0 ± 0
(Bacillota) *Peptoniphilus koenoeneniae*	0 ± 0	0 ± 0	0 ± 0	0 ± 0
(Bacillota) *Peptoniphilus lacrimalis*	0,0123 ± 0,0675	0 ± 0	0 ± 0	0 ± 0
(Bacillota) *Peptoniphilus obesi*	0 ± 0	0 ± 0	0 ± 0	0 ± 0
(Bacillota) *Peptoniphilus tyrrelliae*	0 ± 0	0 ± 0	0 ± 0	0 ± 0
(Bacillota) *Peptostreptococcus anaerobius*	0 ± 0	0,0006 ± 0,0025	0,0006 ± 0,0036	0,0006 ± 0,0036
(Bacillota) *Phascolarctobacterium faecium*	0,1552 ± 0,5021	0,484 ± 0,9071	0,3283 ± 0,5337	0,4975 ± 1,1125
(Bacillota) *Phascolarctobacterium succinatutens*	0,1196 ± 0,3521	0 ± 0	0,0166 ± 0,0912	0,3354 ± 1,179
(Bacillota) *Phocea massiliensis*	0,0485 ± 0,208	0,1773 ± 0,3743	0,1353 ± 0,3295	0,0494 ± 0,12
(Bacillota) *Pseudobutyrivibrio ruminis*	0 ± 0	0 ± 0	0 ± 0	0,0065 ± 0,0356
(Bacillota) *Pseudoflavonifractor capillosus*	0,0044 ± 0,0169	0,037 ± 0,1145	0,0116 ± 0,0293	0,0276 ± 0,0782
(Bacillota) *Pseudoflavonifractor phocaeensis*	0,042 ± 0,1057	0,1943 ± 0,3102	0,1039 ± 0,1412	0,1475 ± 0,3865
(Bacillota) *Pseudoramibacter alactolyticus*	0,0006 ± 0,0036	0 ± 0	0 ± 0	0,001 ± 0,0054
(Bacillota) *Robinsoniella peoriensis*	0,0087 ± 0,0457	0,0533 ± 0,0863	0,0557 ± 0,2759	0,052 ± 0,1431
(Bacillota) *Romboutsia ilealis*	0,021 ± 0,0465	0,0533 ± 0,2883	0 ± 0	0,0006 ± 0,0036
(Bacillota) *Romboutsia lituseburensis*	0 ± 0	0 ± 0	0 ± 0	0 ± 0

(Bacillota) *Romboutsia timonensis*	0,1853 ± 0,6888	2,0496 ± 3,3988	1,0246 ± 1,5143	1,1574 ± 1,454
(Bacillota) *Roseburia faecis*	1,1897 ± 2,6979	0,9796 ± 1,4765	2,1226 ± 2,5096	1,5803 ± 1,9851
(Bacillota) *Roseburia Hominis*	0,215 ± 0,6125	1,3583 ± 2,9281	0,5273 ± 0,6508	0,2443 ± 0,3851
(Bacillota) *Roseburia intestinalis*	0,7324 ± 1,5692	0,861 ± 1,7675	1,1704 ± 2,0722	0,956 ± 1,8774
(Bacillota) *Roseburia inulinivorans*	0,7968 ± 1,7634	0,5136 ± 0,6244	0,414 ± 0,6544	0,6181 ± 1,324
(Bacillota) *Ruminiclostridium cellobioparum*	0 ± 0	0,0006 ± 0,0036	0 ± 0	0 ± 0
(Bacillota) *Ruminiclostridium papyrosolvens*	0 ± 0	0 ± 0	0 ± 0	0 ± 0
(Bacillota) *Ruminiclostridium siraeum*	0,2283 ± 0,8931	0 ± 0	0 ± 0	0,0151 ± 0,0827
(Bacillota) *Ruminiclostridium thermocellum*	0 ± 0	0 ± 0	0 ± 0	0,0066 ± 0,0314
(Bacillota) *Ruminococcus albus*	0,1671 ± 0,6239	1,056 ± 1,356	0,6848 ± 1,39	0,6298 ± 1,1329
(Bacillota) *Ruminococcus bicirculans*	0,232 ± 0,4356	0 ± 0	0,2246 ± 0,8248	0,0326 ± 0,1322
(Bacillota) *Ruminococcus bromii*	7,654 ± 10,0424	3,2686 ± 3,1495	3,6447 ± 4,2482	2,1285 ± 3,6337
(Bacillota) *Ruminococcus callidus*	0,0356 ± 0,1156	0,7 ± 1,2887	0,3899 ± 0,6929	0,6022 ± 1,3465
(Bacillota) *Ruminococcus champanellensis*	0,1397 ± 0,4838	0,393 ± 0,5087	0,3866 ± 0,8518	0,4442 ± 0,9601
(Bacillota) *Ruminococcus faecis*	0 ± 0	0 ± 0	0 ± 0	0 ± 0
(Bacillota) *Ruminococcus flavefaciens*	0,041 ± 0,1574	0,1503 ± 0,4433	0,0416 ± 0,1956	0,0013 ± 0,005
(Bacillota) *Ruminococcus gauvreauii*	0 ± 0	0,0006 ± 0,0036	0 ± 0	0,0345 ± 0,1893
(Bacillota) *Ruminococcus Gauvreauli*	0,0036 ± 0,02	0 ± 0	0 ± 0	0 ± 0
(Bacillota) *Mediterraneibacter gnavus*	0,215 ± 0,5503	0 ± 0	0,2193 ± 0,6211	0,0916 ± 0,3241
(Bacillota) *Ruminococcus gravus*	#DIV/0!	0 ± 0	0 ± 0	0 ± 0
(Bacillota) *Ruminococcus lactaris*	0,1043 ± 0,3557	0,0006 ± 0,0036	0,0886 ± 0,3795	0,1575 ± 0,4679
(Bacillota) *Mediterraneibacter torques*	1,4363 ± 2,2829	0 ± 0	0,1566 ± 0,3645	0,0973 ± 0,3009
(Bacillota) *Ruthenibacterium lactatiformans*	0,037 ± 0,0768	0,109 ± 0,1713	0,0842 ± 0,2026	0,1988 ± 0,7205
(Bacillota) *Saccharofermentans acetigenes*	0 ± 0	0 ± 0	0 ± 0	0 ± 0
(Bacillota) *Sarcina maxima*	0 ± 0	0,0263 ± 0,113	0 ± 0	0 ± 0
(Bacillota) *Selenomonas bovis*	0 ± 0	0,0143 ± 0,0748	0 ± 0	0 ± 0
(Bacillota) *Sellimonas intestinalis*	0,23 ± 0,825	0 ± 0	0 ± 0	0,0073 ± 0,0298
(Bacillota) *Solibacillus isronensis*	0,0005 ± 0,0027	0,0183 ± 0,0878	0,0016 ± 0,0091	0,008 ± 0,0175
(Bacillota) *Solobacterium Moorei*	0 ± 0	0,021 ± 0,0765	0,0026 ± 0,0111	0,0155 ± 0,068
(Bacillota) *Sporobacter termitidis*	0,0809 ± 0,2343	0,3716 ± 0,4107	0,4558 ± 0,8118	0,2192 ± 0,4307
(Bacillota) *Staphylococcus aureus*	0 ± 0	0 ± 0	0 ± 0	0 ± 0
(Bacillota) *Staphylococcus epidermidis*	0 ± 0	0,0026 ± 0,0146	0 ± 0	0 ± 0
(Bacillota) *Staphylococcus equorum*	0,0086 ± 0,0474	0 ± 0	0 ± 0	0 ± 0
(Bacillota) *Staphylococcus piscifermentans*	0 ± 0	0 ± 0	0 ± 0	0 ± 0
(Bacillota) *Staphylococcus pragensis*	0 ± 0	0,0006 ± 0,0036	0 ± 0	0 ± 0
(Bacillota) *Staphylococcus saprophyticus*	0 ± 0	0 ± 0	0 ± 0	0 ± 0
(Bacillota) *Staphylococcus succinus*	0 ± 0	0 ± 0	0 ± 0	0 ± 0
(Bacillota) *Staphylococcus warneri*	0 ± 0	0 ± 0	0 ± 0	0 ± 0

(Bacillota) *Streptococcus agalactiae*	0 ± 0	0 ± 0	0 ± 0	0 ± 0
(Bacillota) *Streptococcus alactolyticus*	0 ± 0	0,001 ± 0,004	0,0356 ± 0,1589	0 ± 0
(Bacillota) *Streptococcus anginosus*	0,1306 ± 0,4541	0,295 ± 0,7627	0 ± 0	0 ± 0
(Bacillota) *Streptococcus australis*	0,0146 ± 0,0766	0,0103 ± 0,044	0,001 ± 0,004	0,0136 ± 0,0748
(Bacillota) *Streptococcus chosunense*	0 ± 0	0 ± 0	0 ± 0	0 ± 0
(Bacillota) *Streptococcus constellatus*	0 ± 0	0 ± 0	0 ± 0	0 ± 0
(Bacillota) *Streptococcus costellatus*	#DIV/0!	0 ± 0	0 ± 0	0 ± 0
(Bacillota) *Streptococcus cristatus*	0,1913 ± 1,0479	0,1916 ± 1,0479	0 ± 0	0 ± 0
(Bacillota) *Streptococcus downei*	0 ± 0	0,0016 ± 0,0091	0 ± 0	0 ± 0
(*Bacillota) Streptococcus dysgalactiae*	0 ± 0	0 ± 0	0 ± 0	0 ± 0
(Bacillota) *Streptococcus equinus*	0 ± 0	0,0116 ± 0,0536	0 ± 0	0,0329 ± 0,1805
(Bacillota) *Streptococcus gallolyticus*	0 ± 0	0 ± 0	0 ± 0	0 ± 0
(Bacillota) *Streptococcus gordonii*	0,041 ± 0,1571	0,0283 ± 0,1514	0,0023 ± 0,0089	0,0023 ± 0,0127
(Bacillota) *Streptococcus gwangjuense*	0 ± 0	0,0016 ± 0,0091	0 ± 0	0 ± 0
(Bacillota) *Streptococcus himalayensis*	0 ± 0	0,0036 ± 0,0088	0,0006 ± 0,0025	0,0006 ± 0,0036
(Bacillota) *Streptococcus infantis*	0 ± 0	0 ± 0	0 ± 0	0 ± 0
(Bacillota) *Streptococcus iniae*	0 ± 0	0 ± 0	0 ± 0	0 ± 0
(Bacillota) *Streptococcus intermedius*	0,0178 ± 0,0974	0,0093 ± 0,0441	0,004 ± 0,0201	0 ± 0
(Bacillota) *Streptococcus koreensis*	0 ± 0	0 ± 0	0 ± 0	0 ± 0
(Bacillota) *Streptococcus lactarius*	0 ± 0	0,002 ± 0,0109	0 ± 0	0 ± 0
(Bacillota) *Streptococcus luteciae*	0 ± 0	0,0056 ± 0,031	0 ± 0	0 ± 0
(Bacillota) *Streptococcus lutetiensis*	0,0136 ± 0,0576	0,7023 ± 2,2334	0 ± 0	0,0006 ± 0,0036
(Bacillota) *Streptococcus massiliensis*	0 ± 0	0 ± 0	0 ± 0	0 ± 0
(Bacillota) *Streptococcus mitis*	0,0906 ± 0,4778	0,104 ± 0,4772	0 ± 0	0,0106 ± 0,0327
(Bacillota) *Streptococcus Mutans*	0,003 ± 0,012	0 ± 0	0 ± 0	0 ± 0
(Bacillota) *Streptococcus oralis*	0,014 ± 0,036	0,0796 ± 0,3673	0,0006 ± 0,0036	0,0016 ± 0,0064
(Bacillota) *Streptococcus parasanguinis*	0,3493 ± 0,6828	0,4273 ± 1,0049	0,005 ± 0,0201	0,0236 ± 0,1122
(Bacillota) *Streptococcus pasteurianus*	0,0003 ± 0,0018	0,0013 ± 0,0043	0,1086 ± 0,5951	0 ± 0
(Bacillota) *Streptococcus Peroris*	0,0673 ± 0,3687	0 ± 0	0 ± 0	0 ± 0
(Bacillota) *Streptococcus plemorphus*	0 ± 0	0 ± 0	0 ± 0	0 ± 0
(Bacillota) *Streptococcus plurextorum*	0 ± 0	0,0003 ± 0,0018	0 ± 0	0 ± 0
(Bacillota) *Streptococcus pseudopneumoniae*	0,001 ± 0,0054	0 ± 0	0 ± 0	0 ± 0
(Bacillota) *Streptococcus rubneri*	0,001 ± 0,0054	0,035 ± 0,1639	0,0249 ± 0,0781	0 ± 0
(Bacillota) *Streptococcus salivarius*	1,2261 ± 3,3345	2,1906 ± 2,5498	0,2193 ± 0,8507	0,2208 ± 0,415
(Bacillota) *Streptococcus saliviloxodontae*	0 ± 0	0,0033 ± 0,0121	0 ± 0	0 ± 0
(Bacillota) *Streptococcus sanguinis*	0,0136 ± 0,066	0,0313 ± 0,0909	0,209 ± 0,6395	0,123 ± 0,6736
(Bacillota) *Streptococcus sinensis*	0 ± 0	0 ± 0	0 ± 0	0 ± 0
(Bacillota) *Streptococcus sobrinus*	0,018 ± 0,0985	0,0016 ± 0,0091	0 ± 0	0 ± 0

(Bacillota) *Streptococcus thermophilus*	0,8863 ± 2,7211	0,1436 ± 0,2572	0,5837 ± 1,0656	0,3959 ± 1,0516
(Bacillota) *Streptococcus troglodytae*	0,0003 ± 0,0018	0,0393 ± 0,0906	0,001 ± 0,0054	0 ± 0
(Bacillota) *Streptococcus vestibularis*	0,0683 ± 0,2192	0,5276 ± 1,4181	0,0023 ± 0,0089	0 ± 0
(Bacillota) *Subdoligranulum variabile*	0 ± 0	0 ± 0	0 ± 0	0,0213 ± 0,117
(Bacillota) *Succiniclasticum ruminis*	0 ± 0	0 ± 0	0 ± 0	0 ± 0
(Bacillota) *Syntrophococcus sucromutans*	0 ± 0	0,0003 ± 0,0018	0 ± 0	0 ± 0
(Bacillota) *Syntrophomonas palmitatica*	0 ± 0	0,0003 ± 0,0018	0 ± 0	0 ± 0
(Bacillota) *Syntrophomonas wolfei*	0 ± 0	0 ± 0	0 ± 0	0,0003 ± 0,0018
(Bacillota) *Tepidibaculum saccharolyticum*	0 ± 0	0,0276 ± 0,144	0,0607 ± 0,2347	0,1131 ± 0,4212
(Bacillota) *Terribacillus halophilus*	0 ± 0	0 ± 0	0 ± 0	0 ± 0
(Bacillota) *Terrisporobacter mayombei*	0,0637 ± 0,2617	0,138 ± 0,3182	0,197 ± 0,6541	0,1826 ± 0,6282
(Bacillota) *Terrisporobacter petrolearius*	0,0003 ± 0,0018	0,0026 ± 0,0063	0,0033 ± 0,0121	0,0016 ± 0,0091
(Bacillota) *Tetragenococcus halophilus*	0 ± 0	0,0106 ± 0,0584	0 ± 0	0,0014 ± 0,0076
(Bacillota) *Tetragenococcus koreensis*	0 ± 0	0,0003 ± 0,0018	0 ± 0	0 ± 0
(Bacillota) *Tissierella creatinini*	0 ± 0	0 ± 0	0 ± 0	0 ± 0
(Bacillota) *Traorella massiliensis*	0 ± 0	0,0003 ± 0,0018	0 ± 0	0 ± 0
(Bacillota) *Turicibacter sanguinis*	0,0876 ± 0,2343	0,1566 ± 0,3599	0,2001 ± 0,4366	0,3327 ± 0,8137
(Bacillota) *Tyzzerella nexilis*	0,005 ± 0,0194	0,0976 ± 0,3026	0,026 ± 0,1368	0,0122 ± 0,0497
(Bacillota) *Vagococcus martis*	0 ± 0	0,0026 ± 0,0146	0 ± 0	0 ± 0
(Bacillota) *Vallitalea pronyensis*	0 ± 0	0,001 ± 0,0054	0 ± 0	0 ± 0
(Bacillota) *Veillonella alcalescens*	0 ± 0	0,0006 ± 0,0036	0 ± 0	0 ± 0
(Bacillota) *Veillonella atypica*	0,0086 ± 0,0308	0,014 ± 0,0349	0 ± 0	0,0082 ± 0,0409
(Bacillota) *Veillonella dispar*	0,0036 ± 0,0124	0,0053 ± 0,0183	0,0046 ± 0,0255	0,0026 ± 0,0146
(Bacillota) *Veillonella infantium*	0 ± 0	0 ± 0	0 ± 0	0 ± 0
(Bacillota) *Veillonella parvula*	0,144 ± 0,6485	0,0386 ± 0,0832	0,017 ± 0,061	0,004 ± 0,0219
(Bacillota) *Veillonella tobetsuensis*	0 ± 0	0,0026 ± 0,0146	0 ± 0	0 ± 0
(Bacillota) *Weissella cibaria*	0 ± 0	0,0066 ± 0,0311	0 ± 0	0 ± 0
(Bacillota) *Weissella confusa*	0 ± 0	0,0376 ± 0,1433	0,0663 ± 0,3633	0 ± 0
(Bacillota) *Weissella viridescens*	0,0016 ± 0,0091	0,0013 ± 0,0073	0 ± 0	0 ± 0
(Bacillota) *macedonicus*	0 ± 0	0,0656 ± 0,3596	0 ± 0	0 ± 0
(Bacillota) *Veillonella rogosae*	0 ± 0	0 ± 0	0 ± 0	0,0006 ± 0,0036
(Fusobacteriota) *Cetobacterium somerae*	0,0436 ± 0,2391	0 ± 0	0 ± 0	0 ± 0
(Fusobacteriota) *Fusobacterium equinum*	0 ± 0	0 ± 0	0 ± 0	0 ± 0
(Fusobacteriota) *Fusobacterium nucleatum*	0 ± 0	0 ± 0	0 ± 0	0,0027 ± 0,0149
(Fusobacteriota) *Fusobacterium varium*	0,0744 ± 0,4075	0 ± 0	0 ± 0	0 ± 0
(Fusobacteriota) *Sneathia sanguinegens*	0 ± 0	0 ± 0	0 ± 0	0 ± 0
(Bacillota) *Clostridium phocaeense*	0,027 ± 0,1478	0,0393 ± 0,2154	0 ± 0	0 ± 0
(Lentisphaereae) *Victivallis vadensis*	0,0064 ± 0,0354	0,063 ± 0,2974	0,0212 ± 0,0705	0,0163 ± 0,0875

(Lentisphaereae) *Oligosphaera ethanolica*	0 ± 0	0 ± 0	0 ± 0	0 ± 0
(Methanobacteriota) *Methanobrevibacter smithii*	0,5834 ± 1,0101	0,1646 ± 0,2985	0,0722 ± 0,1684	0,1136 ± 0,3539
(Methanobacteriota) *Methanomassiliicoccus intestinalis*	0 ± 0	0 ± 0	0 ± 0	0,0006 ± 0,0036
(Methanobacteriota) *Methanosphaera stadtmanae*	0,0213 ± 0,0955	0,0076 ± 0,0338	0 ± 0	0 ± 0
(Mycoplasmatota) *Anaeroplasma varium*	0 ± 0	0,0043 ± 0,0237	0 ± 0	0 ± 0
(Mycoplasmatota) *Asteroleplasma anaerobium*	0 ± 0	0 ± 0	0 ± 0	0 ± 0
(Mycoplasmatota) *Haloplasma contractile*	0,004 ± 0,022	0,0013 ± 0,0057	0,0013 ± 0,0073	0 ± 0
(Mycoplasmatota) *Mycoplasma hominis*	0 ± 0	0 ± 0	0 ± 0	0 ± 0
(Mycoplasmatota) *Spiroplasma alleghenense*	0 ± 0	0 ± 0	0 ± 0	0 ± 0
(Mycoplasmatota) *Spiroplasma phoeniceum*	0,0027 ± 0,0151	0 ± 0	0 ± 0	0 ± 0
(Planctomycetota) *Mariniblastus fucicola*	0 ± 0	0 ± 0	0 ± 0	0,0415 ± 0,2273
(Planctomycetota) *Pirellula staleyi*	0 ± 0	0 ± 0	0 ± 0	0 ± 0
(Pseudomonadota) *Acinetobacter baumannii*	0 ± 0	0 ± 0	0 ± 0	0 ± 0
(Pseudomonadota) *Acinetobacter bouvetii*	0 ± 0	0 ± 0	0 ± 0	0 ± 0
(Pseudomonadota) *Aeromonas jandae*	0 ± 0	0 ± 0	0 ± 0	0 ± 0
(Pseudomonadota) *Aestuariispira insulae*	0 ± 0	0,0096 ± 0,0493	0,007 ± 0,0215	0,0199 ± 0,0787
(Pseudomonadota) *Aggregatibacter aphrophilus*	0 ± 0	0 ± 0	0 ± 0	0 ± 0
(Pseudomonadota) *Aggregatibacter segnis*	0,0003 ± 0,0018	0 ± 0	0 ± 0	0 ± 0
(Pseudomonadota) *Aliidongia dinghuensis*	0 ± 0	0,0006 ± 0,0036	0 ± 0	0 ± 0
(Pseudomonadota) *Anaerobiospirillum succiniciproducens*	0 ± 0	0 ± 0	0 ± 0	0 ± 0
(Pseudomonadota) *Anaerobiospirillum thomasii*	0 ± 0	0,054 ± 0,2901	0,2649 ± 1,0381	0 ± 0
(Pseudomonadota) *Azomonas agilis*	0 ± 0	0,0003 ± 0,0018	0 ± 0	0 ± 0
(Pseudomonadota) *Azospirillum lipoferum*	0 ± 0	0 ± 0	0 ± 0	0 ± 0
(Pseudomonadota) *Azotobacter chroococcum*	0 ± 0	0 ± 0	0 ± 0	0 ± 0
(Pseudomonadota) *Bilophila wadsworthia*	0 ± 0	0 ± 0	0 ± 0	1,5454 ± 4,7988
(Pseudomonadota) *Buttiauxella agrestis*	0 ± 0	0 ± 0	0 ± 0	0 ± 0
(Pseudomonadota) *Buttiauxella noackiae*	0,0008 ± 0,0043	0 ± 0	0 ± 0	0 ± 0
(Pseudomonadota) *Campylobacter gracilis*	0 ± 0	0 ± 0	0 ± 0	0 ± 0
(Pseudomonadota) *Campylobacter hominis*	0,0426 ± 0,2336	0 ± 0	0 ± 0	0 ± 0
(Pseudomonadota) *Campylobacter hyointestinalis*	0 ± 0	0,0006 ± 0,0036	0 ± 0	0 ± 0
(Pseudomonadota) *Campylobacter mucosalis*	0 ± 0	0,0003 ± 0,0018	0 ± 0	0 ± 0
(Pseudomonadota) *Campylobacter rectus*	0 ± 0	0 ± 0	0 ± 0	0 ± 0
(Pseudomonadota) *Campylobacter sputorum*	0 ± 0	0 ± 0	0 ± 0	0 ± 0
(Pseudomonadota) *Campylobacter ureolyticus*	0,0016 ± 0,0091	0 ± 0	0 ± 0	0 ± 0
(Pseudomonadota) *Cardiobacterium hominis*	0 ± 0	0 ± 0	0 ± 0	0 ± 0
(Pseudomonadota) *Cedecea lapagei*	0 ± 0	0 ± 0	0 ± 0	0 ± 0
(Pseudomonadota) *Citrobacter amalonaticus*	0 ± 0	0 ± 0	0 ± 0	0 ± 0
(Pseudomonadota) *Citrobacter braakii*	0 ± 0	0 ± 0	0 ± 0	0 ± 0

(Pseudomonadota) *Citrobacter cronae*	0 ± 0	0 ± 0	0 ± 0	0 ± 0
(Pseudomonadota) *Citrobacter freundii*	0 ± 0	0,0083 ± 0,0369	0,0543 ± 0,2975	0 ± 0
(Pseudomonadota) *Citrobacter Gillenii*	0 ± 0	0 ± 0	0 ± 0	0 ± 0
(Pseudomonadota) *Citrobacter koseri*	0 ± 0	0,088 ± 0,4819	0 ± 0	0,001 ± 0,0054
(Pseudomonadota) *Citrobacter murliniae*	0 ± 0	0,005 ± 0,0273	0 ± 0	0 ± 0
(Pseudomonadota) *Citrobacter werkmanii*	0 ± 0	0 ± 0	0 ± 0	0 ± 0
(Pseudomonadota) *Citrobacter youngae*	0 ± 0	0 ± 0	0 ± 0	0 ± 0
(Pseudomonadota) *Comamonas aquatica*	0 ± 0	0 ± 0	0 ± 0	0 ± 0
(Pseudomonadota) *Comamonas jiangduensis*	0 ± 0	0 ± 0	0 ± 0	0 ± 0
(Pseudomonadota) *Comamonas kerstersii*	0 ± 0	0 ± 0	0 ± 0	0 ± 0
(Pseudomonadota) *Comamonas terrae*	0 ± 0	0 ± 0	0 ± 0	0 ± 0
(Pseudomonadota) *Comamonas terrigena*	0 ± 0	0 ± 0	0 ± 0	0 ± 0
(Pseudomonadota) *Cronobacter dublinensis*	0 ± 0	0,0006 ± 0,0036	0 ± 0	0 ± 0
(Pseudomonadota) *Cronobacter muytjensii*	0 ± 0	0 ± 0	0 ± 0	0 ± 0
(Pseudomonadota) *Cronobacter sakazakii*	0 ± 0	0 ± 0	0 ± 0	0 ± 0
(Pseudomonadota) *Cronobacter turicensis*	0 ± 0	0,0016 ± 0,0091	0 ± 0	0,0464 ± 0,2541
(Pseudomonadota) *Cupidesulfovibrio oxamicus*	0,0442 ± 0,2077	0,0243 ± 0,0554	0,0093 ± 0,0316	0,0153 ± 0,0486
(Pseudomonadota) *Dakarella massiliensis*	0 ± 0	0 ± 0	0 ± 0	0 ± 0
(Pseudomonadota) *Desulfocella halophila*	0 ± 0	0 ± 0	0 ± 0	0 ± 0
(Pseudomonadota) *Desulfosarcina alkanivorans*	0 ± 0	0,003 ± 0,0164	0 ± 0	0 ± 0
(Pseudomonadota) *Desulfovibrio alcoholivorans*	0 ± 0	0 ± 0	0 ± 0	0 ± 0
(Pseudomonadota) *Desulfovibrio desulfuricans*	0,0078 ± 0,035	0,0986 ± 0,1682	0,059 ± 0,1903	0,4771 ± 1,6184
(Pseudomonadota) *Desulfovibrio fairfieldensis*	0,0066 ± 0,0365	0 ± 0	0 ± 0	0 ± 0
(Pseudomonadota) *Desulfovibrio intestinalis*	0 ± 0	0 ± 0	0 ± 0	0 ± 0
(Pseudomonadota) *Desulfovibrio legallii*	0 ± 0	0 ± 0	0 ± 0	0 ± 0
(Pseudomonadota) *Desulfovibrio piger*	0,0023 ± 0,0089	0,0393 ± 0,101	0,0835 ± 0,187	0,0409 ± 0,1093
(Pseudomonadota) *Desulfovibrio simplex*	0 ± 0	0,0003 ± 0,0018	0 ± 0	0,0006 ± 0,0034
(Pseudomonadota) *Desulfovibrio vulgaris*	0 ± 0	0,0016 ± 0,0091	0 ± 0	0 ± 0
(Pseudomonadota) *Duodenibacillus massiliensis*	0,0296 ± 0,1235	0,034 ± 0,1603	0,0148 ± 0,0521	0,0044 ± 0,0208
(Pseudomonadota) *Edwardsiella hoshinae*	0 ± 0	0 ± 0	0 ± 0	0 ± 0
(Pseudomonadota) *Enterobacter cancerogenus*	0 ± 0	0 ± 0	0 ± 0	0 ± 0
(Pseudomonadota) *Enterobacter cloacae*	0 ± 0	0,0003 ± 0,0018	0,004 ± 0,0161	0,054 ± 0,2056
(Pseudomonadota) *Enterobacter hormaechei*	0 ± 0	0 ± 0	0 ± 0	0 ± 0
(Pseudomonadota) *Enterobacter kobei*	0 ± 0	0 ± 0	0 ± 0	0 ± 0
(Pseudomonadota) *Enterobacter ludwigii*	0 ± 0	0,0063 ± 0,0346	0 ± 0	0 ± 0
(Pseudomonadota) *Enterobacter mori*	0 ± 0	0 ± 0	0,0006 ± 0,0036	0,0053 ± 0,0292
(Pseudomonadota) *Enterobacter sacchari*	0 ± 0	0 ± 0	0 ± 0	0 ± 0
(Pseudomonadota) *Enterobacter soli*	0,0172 ± 0,0945	0,0073 ± 0,0401	0 ± 0	0 ± 0

(Pseudomonadota) *Erwinia persicina*	0 ± 0	0 ± 0	0 ± 0	0 ± 0
(Pseudomonadota) *Erwinia rhapontici*	0 ± 0	0 ± 0	0 ± 0	0 ± 0
(Pseudomonadota) *Erwinia toletana*	0 ± 0	0 ± 0	0 ± 0	0 ± 0
(Pseudomonadota) *Escherichia albertii*	0 ± 0	0 ± 0	0 ± 0	0 ± 0
(Pseudomonadota) *Escherichia coli*	4,6489 ± 13,3352	0,189 ± 0,6554	0,7957 ± 3,428	0,21 ± 0,8674
(Pseudomonadota) *Escherichia marmotae*	0,0442 ± 0,1813	0,777 ± 1,9615	0,1988 ± 0,9022	0,02 ± 0,0691
(Pseudomonadota) *Fodinicurvata fenggangensis*	0 ± 0	0 ± 0	0 ± 0	0 ± 0
(Pseudomonadota) *Fodinicurvata sediminis*	0 ± 0	0,0006 ± 0,0036	0 ± 0	0 ± 0
(Pseudomonadota) *Gemmiger formicilis*	0,3053 ± 0,4555	0 ± 0	0,0453 ± 0,1168	0,0363 ± 0,1205
(Pseudomonadota) *Geobacter pickeringii*	0 ± 0	0 ± 0	0 ± 0	0 ± 0
(Pseudomonadota) *Haemophilus parainfluenzae*	0,002 ± 0,0109	0,0183 ± 0,0875	0,0357 ± 0,1061	0,0251 ± 0,0779
(Pseudomonadota) *Haemophilus pittmaniae*	0 ± 0	0 ± 0	0 ± 0	0 ± 0
(Pseudomonadota) *Hafnia paralvei*	0 ± 0	0,0193 ± 0,0536	0 ± 0	0 ± 0
(Pseudomonadota) *Hellea balneolensis*	0 ± 0	0 ± 0	0 ± 0	0 ± 0
(Pseudomonadota) *Henriciella pelagia*	0 ± 0	0 ± 0	0 ± 0	0,0353 ± 0,1935
(Pseudomonadota) *Hwanghaeella grinnelliae*	0 ± 0	0,0023 ± 0,011	0 ± 0	0 ± 0
(Pseudomonadota) *Insolitispirillum peregrinum*	0 ± 0	0 ± 0	0 ± 0	0 ± 0
(Pseudomonadota) *Kingella oralis*	0 ± 0	0 ± 0	0 ± 0	0 ± 0
(Pseudomonadota) *Klebsiella michiganensis*	0,057 ± 0,3122	0 ± 0	0 ± 0	0 ± 0
(Pseudomonadota) *Klebsiella oxytoca*	0,0033 ± 0,0182	0,001 ± 0,0054	0 ± 0	0,0003 ± 0,0018
(Pseudomonadota) *Klebsiella pneumoniae*	1,4462 ± 3,8111	0,1156 ± 0,3708	0,172 ± 0,7943	0,0016 ± 0,0091
(Pseudomonadota) *Klebsiella quasipneumoniae*	0 ± 0	0 ± 0	0,071 ± 0,374	0 ± 0
(Pseudomonadota) *Klebsiella variicola*	0,711 ± 2,6609	0,0233 ± 0,1278	0 ± 0	0 ± 0
(Pseudomonadota) *Kluyvera cryocrescens*	0,0206 ± 0,1131	0 ± 0	0 ± 0	0 ± 0
(Pseudomonadota) *Kordiimonas aquimaris*	0 ± 0	0,009 ± 0,0492	0,004 ± 0,0154	0,0149 ± 0,0779
(Pseudomonadota) *Kosakonia oryzae*	0 ± 0	0 ± 0	0 ± 0	0 ± 0
(Pseudomonadota) *Lautropia mirabilis*	0,0003 ± 0,0018	0 ± 0	0 ± 0	0 ± 0
(Pseudomonadota) *Leclercia adecarboxylata*	0 ± 0	0 ± 0	0 ± 0	0,0026 ± 0,0146
(Pseudomonadota) *Limnobacter litoralis*	0 ± 0	0,0013 ± 0,0057	0 ± 0	0 ± 0
(Pseudomonadota) *Lysobacter tolerans*	0 ± 0	0 ± 0	0 ± 0	0 ± 0
(Pseudomonadota) *Mailhella massiliensis*	0 ± 0	0,0063 ± 0,0179	0,005 ± 0,0153	0,0767 ± 0,3141
(Pseudomonadota) *Mannheimia varigena*	0 ± 0	0 ± 0	0 ± 0	0 ± 0
(Pseudomonadota) *Marivibrio halodurans*	0 ± 0	0 ± 0	0 ± 0	0,0063 ± 0,0346
(Pseudomonadota) *Massilia timonae*	0 ± 0	0 ± 0	0 ± 0	0 ± 0
(Pseudomonadota) *Mesosutterella multiformis*	0,0003 ± 0,0018	0 ± 0	0,0073 ± 0,0289	0 ± 0
(Pseudomonadota) *Methylobacter luteus*	0 ± 0	0 ± 0	0 ± 0	0 ± 0
(Pseudomonadota) *Mixta theicola*	0 ± 0	0 ± 0	0 ± 0	0 ± 0
(Pseudomonadota) *Morganella morganii*	0 ± 0	0,0003 ± 0,0018	0,0058 ± 0,0319	0 ± 0

(Pseudomonadota) *Neisseria macacae*	0 ± 0	0 ± 0	0 ± 0	0 ± 0
(Pseudomonadota) *Neisseria oralis*	0 ± 0	0 ± 0	0 ± 0	0 ± 0
(Pseudomonadota) *Nicoletella semolina*	0 ± 0	0 ± 0	0 ± 0	0 ± 0
(Pseudomonadota) *Nitratifractor salsuginis*	0 ± 0	0,0003 ± 0,0018	0,005 ± 0,0169	0,006 ± 0,0228
(Pseudomonadota) *Obesumbacterium proteus*	0 ± 0	0 ± 0	0 ± 0	0 ± 0
(Pseudomonadota) *Oxalobacter formigenes*	0 ± 0	0,0056 ± 0,0259	0,0006 ± 0,0036	0 ± 0
(Pseudomonadota) *Pantoea agglomerans*	0,0166 ± 0,0909	0,0043 ± 0,0237	0,0628 ± 0,3439	0 ± 0
(Pseudomonadota) *Pantoea allii*	0 ± 0	0 ± 0	0 ± 0	0 ± 0
(Pseudomonadota) *Pantoea dispersa*	0 ± 0	0 ± 0	0 ± 0	0 ± 0
(Pseudomonadota) *Paracoccus carotinifaciens*	0,0023 ± 0,0129	0 ± 0	0,0003 ± 0,0018	0,0003 ± 0,0018
(Pseudomonadota) *Paracoccus lutimaris*	0 ± 0	0 ± 0	0 ± 0	0 ± 0
(Pseudomonadota) *Parasutterella excrementihominis*	0,0006 ± 0,0036	0 ± 0	0,0093 ± 0,0426	0,0046 ± 0,0255
(Pseudomonadota) *Parasutterella secunda*	0,0013 ± 0,0073	0 ± 0	0 ± 0	0,0203 ± 0,1111
(Pseudomonadota) *Pelagibius litoralis*	0 ± 0	0 ± 0	0 ± 0	0 ± 0
(Pseudomonadota) *Pelomonas saccharophila*	0 ± 0	0 ± 0	0 ± 0	0 ± 0
(Pseudomonadota) *Planctobacterium marinum*	0 ± 0	0 ± 0	0 ± 0	0 ± 0
(Pseudomonadota) *Pluralibacter pyrinus*	0 ± 0	0 ± 0	0 ± 0	0 ± 0
(Pseudomonadota) *Providencia stuartii*	0 ± 0	0 ± 0	0 ± 0	0 ± 0
(Pseudomonadota) *Pseudescherichia vulneris*	0,0586 ± 0,3213	0 ± 0	0 ± 0	0 ± 0
(Pseudomonadota) *Quisquiliibacterium transsilvanicum*	0 ± 0	0,001 ± 0,004	0 ± 0	0 ± 0
(Pseudomonadota) *Raoultella ornithinolytica*	0 ± 0	0,009 ± 0,0492	0 ± 0	0 ± 0
(Pseudomonadota) *Raoultella planticola*	0 ± 0	0,0093 ± 0,0511	0 ± 0	0 ± 0
(Pseudomonadota) *Rhodobium orientis*	0 ± 0	0 ± 0	0,0004 ± 0,0023	0 ± 0
(Pseudomonadota) *Rhodoferax ferrireducens*	0 ± 0	0 ± 0	0 ± 0	0 ± 0
(Pseudomonadota) *Rhodospirillum rubrum*	0 ± 0	0 ± 0	0 ± 0	0 ± 0
(Pseudomonadota) *Salmonella bongori*	0 ± 0	0 ± 0	0 ± 0	0 ± 0
(Pseudomonadota) *Salmonella enterica*	0 ± 0	0 ± 0	0 ± 0	0 ± 0
(Pseudomonadota) *Serratia marcescens*	0 ± 0	0 ± 0	0 ± 0	0 ± 0
(Pseudomonadota) *Serratia nematodiphila*	0 ± 0	0 ± 0	0 ± 0	0 ± 0
(Pseudomonadota) *Serratia rubidaea*	0 ± 0	0 ± 0	0 ± 0	0 ± 0
(Pseudomonadota) *Serratia symbiotica*	0 ± 0	0 ± 0	0 ± 0	0,0009 ± 0,0049
(Pseudomonadota) *Shigella boydii*	0,0016 ± 0,0091	0,0106 ± 0,0378	0,0006 ± 0,0036	0,004 ± 0,0219
(Pseudomonadota) *Shigella dysenteriae*	0 ± 0	0,7536 ± 2,0822	0 ± 0	0 ± 0
(Pseudomonadota) *Shigella flexneri*	0,0316 ± 0,1247	0,0486 ± 0,1216	0,0603 ± 0,3174	0 ± 0
(Pseudomonadota) *Shimwellia blattae*	0 ± 0	0 ± 0	0 ± 0	0 ± 0
(Pseudomonadota) *Shinella zoogloeoides*	0 ± 0	0 ± 0	0 ± 0	0 ± 0
(Pseudomonadota) *Stenotrophomonas maltophilia*	0 ± 0	0 ± 0	0 ± 0	0 ± 0
(Pseudomonadota) *Succinivibrio dextrinosolvens*	0 ± 0	0 ± 0	0 ± 0	0 ± 0

(Pseudomonadota) *Sulfurovum aggregans*	0 ± 0	0 ± 0	0 ± 0	0 ± 0
(Pseudomonadota) *Sutterella faecalis*	0 ± 0	0 ± 0	0,0013 ± 0,0073	0 ± 0
(Pseudomonadota) *Sutterella massiliensis*	0,0434 ± 0,1375	0,054 ± 0,0992	0,005 ± 0,0179	0,0004 ± 0,0021
(Pseudomonadota) *Sutterella parvirubra*	0 ± 0	0 ± 0	0,013 ± 0,0713	0 ± 0
(Pseudomonadota) *Sutterella stercoricanis*	0 ± 0	0 ± 0	0,004 ± 0,0219	0 ± 0
(Pseudomonadota) *Sutterella Timonensis*	0,0203 ± 0,1111	0,0893 ± 0,4744	0,0538 ± 0,2846	0,192 ± 0,4526
(Pseudomonadota) *Sutterella wadsworthensis*	0,017 ± 0,0771	0,0193 ± 0,0631	0,4211 ± 0,652	0,4652 ± 0,7814
(Pseudomonadota) *Suttonella indologenes*	0 ± 0	0 ± 0	0 ± 0	0 ± 0
(Pseudomonadota) *Syntrophorhabdus aromaticivorans*	0 ± 0	0 ± 0	0 ± 0	0 ± 0
(Pseudomonadota) *Thiobacillus denitrificans*	0 ± 0	0 ± 0	0 ± 0	0 ± 0
(Pseudomonadota) *Trabulsiella odontotermitis*	0 ± 0	0,0003 ± 0,0018	0 ± 0	0 ± 0
(Pseudomonadota) *Turicimonas muris*	0,0632 ± 0,3176	0,1283 ± 0,2986	0,0245 ± 0,0516	0,0944 ± 0,2472
(Pseudomonadota) *Yersinia pseudotuberculosis*	0 ± 0	0,4816 ± 2,6381	0 ± 0	0 ± 0
(Pseudomonadota) *Yokenella regensburgei*	0 ± 0	0 ± 0	0 ± 0	0 ± 0
(Spirochaetota) *Brachyspira hyodysenteriae*	0 ± 0	0 ± 0	0 ± 0	0 ± 0
(Spirochaetota) *Brachyspira intermedia*	0 ± 0	0 ± 0	0 ± 0	0 ± 0
(Spirochaetota) *Treponema succinifaciens*	0 ± 0	0 ± 0	0 ± 0	0 ± 0
(Synergistetes) *Cloacibacillus evryensis*	0,0108 ± 0,0591	0,002 ± 0,008	0 ± 0	0,0003 ± 0,0018
(Synergistetes) *Cloacibacillus porcorum*	0 ± 0	0 ± 0	0 ± 0	0,001 ± 0,0058
(Synergistota) *Pyramidobacter piscolens*	0 ± 0	0 ± 0	0 ± 0	0 ± 0
(Verrucomicrobiota) *Akkermansia muciniphila*	0,9996 ± 2,1782	0,56 ± 1,203	0,6553 ± 1,2656	0,447 ± 1,06
(Verrucomicrobiota) *Cerasicoccus frondis*	0 ± 0	0 ± 0	0 ± 0	0 ± 0
(Verrucomicrobiota) *Coraliomargarita sinensis*	0 ± 0	0,02 ± 0,0834	0 ± 0	0 ± 0
(Verrucomicrobiota) *Rubritalea profundi*	0 ± 0	0 ± 0	0 ± 0	0 ± 0
(Verrucomicrobiota) *Ruficoccus amylovorans*	0 ± 0	0,0093 ± 0,0511	0 ± 0	0,0013 ± 0,0073

Values are expressed as mean and standard deviation (M ± SD) for continuous variables.
The taxonomy reports the generic name and specific epithet, while the phylum to which the species belongs is reported in parentheses.
